# A Spectroscopic Criterion for Identifying the Degree of Ground-Level Near-Degeneracy Derived from Effective Hamiltonian Analyses of Three-Coordinate Iron Complexes

**DOI:** 10.1021/jacsau.4c01256

**Published:** 2025-02-06

**Authors:** Wang Chen, Nikolai Kochetov, Thomas Lohmiller, Qing Liu, Liang Deng, Alexander Schnegg, Shengfa Ye

**Affiliations:** † State Key Laboratory of Catalysis, Dalian Institute of Chemical Physics, Chinese Academy of Sciences, 457 Zhongshan Road, Dalian 116023, China; ‡ EPR Research Group, Max Planck Institute for Chemical Energy Conversion, D-45470 Mülheim an der Ruhr, Germany; § EPR4Energy Joint Lab, Department Spins in Energy Conversion and Quantum Information Science, Helmholtz-Zentrum Berlin für Materialien und Energie GmbH, 12489 Berlin, Germany; ∥ Institut für Chemie, Humboldt–Universität zu Berlin, 12489 Berlin, Germany; ⊥ State Key Laboratory of Organometallic Chemistry, Shanghai Institute of Organic Chemistry, University of Chinese Academy of Sciences, Chinese Academy of Sciences, Shanghai 200032, China; # University of Chinese Academy of Sciences, Beijing 100049, China; ∇ Key Laboratory of Bioinorganic and Synthetic Chemistry of Ministry of Education, Guangdong Basic Research Center of Excellence for Functional Molecular Engineering, School of Chemistry, IGCME, Sun Yat-Sen University, Guangzhou 510275, China

**Keywords:** low-coordinate complexes, orbitally near-degenerate ground level, magnetometry, THz-EPR spectroscopy, zero-field splitting, *g*-tensor, magneto-structural correlation, spin−orbit coupling

## Abstract

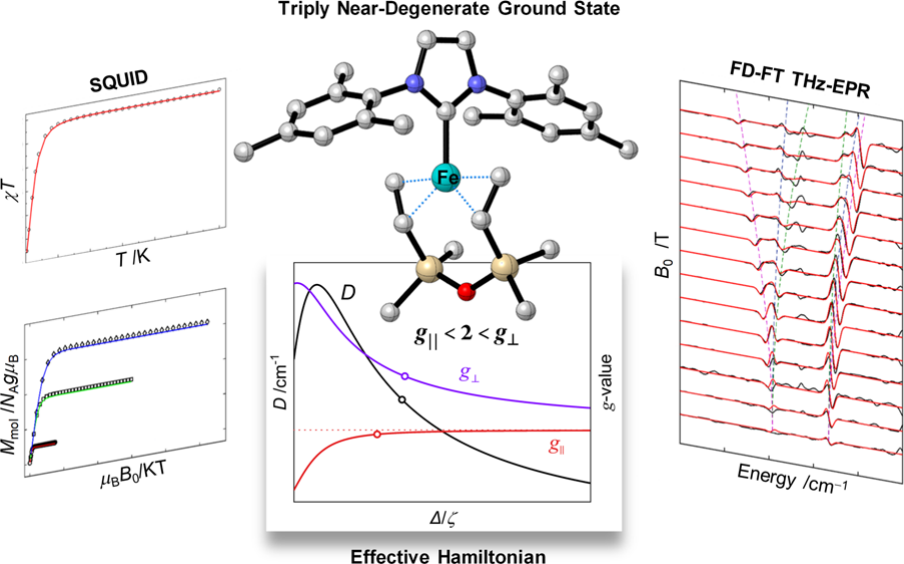

The fascinating magnetic and catalytic properties of coordinatively unsaturated 3d metal complexes are a manifestation of their electronic structures, in particular their nearly doubly or triply degenerate orbital ground levels. Here, we propose a criterion to determine the degree of degeneracy of this class of complexes based on their experimentally accessible magnetic anisotropy (parametrized by the electron spin *g*- and zero-field splitting (ZFS)-tensors). The criterion is derived from a comprehensive spectroscopic and theoretical study in the trigonal planar iron(0) complex, [(IMes)Fe(dvtms)] (IMes = 1,3-di(2′,4′,6′-trimethylphenyl)imidazol-2-ylidene, dvtms = divinyltetramethyldisiloxane, **1**). Accurate ZFS-values (*D* = +33.54 cm^–1^, *E*/*D* = 0.09) and *g*-values (*g*_∥_ = 1.96, *g*_⊥_ = 2.45) of the triplet (*S* = 1) ground level of complex **1** were determined by complementary THz-EPR spectroscopy and SQUID magnetometry. In-depth effective Hamiltonian (EH) analyses coupled to wave-function-based *ab initio* calculations show that **1** features a ground level with three energetically close-lying orbital states with a “two-above-one” energy pattern. The observed magnetic anisotropy results from mixing of the two excited electronic states with the ground state by spin–orbit coupling (SOC). EH investigations on **1** and related complexes allowed us to generalize this finding and establish the anisotropy of the ***g***- and ZFS-tensors as spectroscopic markers for assigning two- or three-fold orbital near-degeneracy.

## Introduction

**Chart 1:**
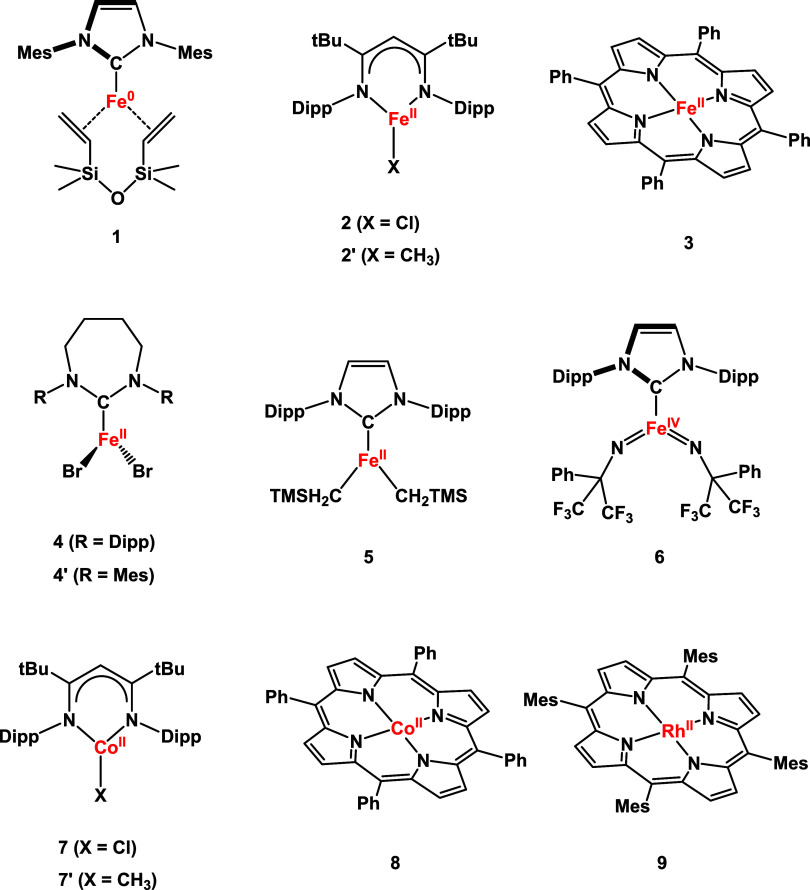
Low-Coordinate Complexes under Investigation

Transition-metal complexes with unsaturated coordination are intensively studied for their role as catalytic active sites and pivotal reaction intermediates in demanding chemical transformations. Isolated low-coordinate metal atoms dispersed on supports as single-atom catalysts have been reported widely to achieve high activity and selectivity.^1^ In biology, N_2_ binding and subsequent functionalization has been proposed to take place at the three-coordinate iron centers of the iron–molybdenum cofactor of nitrogenase.^2^ In synthetic chemistry, reduction of three-coordinate iron(II) chloride complex [LFe^II^Cl] (**2** in Chart 1, L = HC[C(^*t*^Bu)N(2,6-iPr_2_C_6_H_3_)]^2–^) gives dinitrogen complex K_2_[LFeNNFeL] and ultimately results in N–N bond cleavage.^3^ Interest in three-coordinate iron(0) complexes was sparked by their successful application as catalysts for C–C bond formation reactions. These include the catalytic cross-electrophile coupling of aryl chlorides with unactivated alkyl chlorides,^4^ stereoselective C–H alkylation reaction of indole derivatives,^5^ and the Suzuki biaryl coupling of aryl chloride substrates with activated aryl boronic esters.^6^ Furthermore, it has been shown that square-planar [Fe^II^(TPP)] (**3**, TPP^2–^ = tetraphenylporphyrinate dianion) and its derivatives exhibit one of the highest homogeneous catalytic performances for electrochemical CO_2_ reduction.^7^ Combined spectroscopic and computational investigations have shown that three close lying orbital states contribute to the ground level of **3**.^8,9^ Further detailed analysis of the structure–function relationship identified this extraordinary electronic structure as a key factor for the extensive CO_2_ reduction activity of complex **3**.^10^ Hereafter, for clarity we differentiate the terms level and state, in that an energy level of a system with orbital degeneracy may possess several different constituent orbital states.

Energetically almost degenerate d orbitals are a consequence of the very weak ligand fields in coordinatively unsaturated 3d complexes. The resulting electronic structures not only determine their chemical properties, but also their often very large magnetic anisotropies. The latter property renders them potential building blocks for the synthesis of molecular nanomagnets.^11^ As typical examples, a series of two-coordinate high-spin Fe(I/II)^12^ and Co(I/II)^13^ complexes were identified as single-molecule magnets with very large magnetic anisotropies, which has been traced back to the presence of two-fold near-degeneracy in the ground level.

Three-coordinate complexes can also exhibit pronounced magnetic anisotropies, but correlation with their structures is less obvious at first sight. For instance, trigonal planar high-spin iron(II) complexes [Fe(7-Dipp)Br_2_] (7-Dipp = 1,3-bis(2,6-diisopropylphenyl)-4,5,6,7-tetrahydro-[1,3]-diazepin-2-ylidene, **4**), [Fe(7-Mes)Br_2_] (7-Mes = 1,3-bis(2,4,6-trimethylphenyl)-4,5,6,7-tetrahydro-[1,3]-diazepin-2-ylidene, **4**′) and [(IPr)Fe(CH_2_TMS)_2_] (IPr = 1,3-bis(2′,6′-diisopropylphenyl)imidazol-2-ylidene, CH_2_TMS = bis(trimethylsilyl)methyl, **5**) (Chart 1), all stabilized by a cyclic NHC ligand, share similar geometric structures but exhibit disparate magnetic properties. Easy-plane magnetic anisotropy is found for **4** and **4**′, while **5** features eas*y*-axis magnetic anisotropy.^14^ As elaborated below, the differing magnetic anisotropies measured for complexes **4**, **4**′ and **5** result from the distinct orbital near-degeneracy of their ground levels.

Undoubtedly, both chemical activities and magnetic properties of coordinatively unsaturated metal complexes are determined by their peculiar electronic structures, more precisely, critically hinged on the degree of orbital degeneracy of their ground levels. In principle, exact orbital degeneracy cannot exist, because inevitable Jahn–Teller or Renner–Teller distortions will lift the orbital degeneracy and hence the resulting state degeneracy to a certain extent.^15^ Concurrently, spin–orbit coupling (SOC) within this manifold of pseudodegenerate orbital states, if present, tends to restore the orbital degeneracy and gives rise to first-order orbital angular momentum in the ground level.

In earlier work,^16^ orbitally near-degenerate systems were proposed to be distinguished by Δ*E* < 10ζ, where Δ*E* is the nonrelativistic energy separation between the ground and the lowest-energy d-d excited state, and ζ the effective one-electron SOC constant of the metal center. It follows from this criterion that, even under a zeroth-order approximation, the SOC among different components of orbital states in the ground level has to be treated on an equal footing with the ligand field splitting, since both interactions are of the same order of magnitude. In contrast, for orbitally nondegenerate complexes, the SOC between the ground and excited levels can be adequately approximated as a perturbation, as well-documented in the literature.^17^

Strictly speaking, Δ*E* is not an experimental observable, because the associated ligand-field electronic transition measured experimentally includes SOC effects. Even so, such a transition is, however, intrinsically electric dipole forbidden. Thus, its low intensity renders the detection difficult in the near-infrared region (Δ*E* < 10^4^ cm^–1^), considering the magnitude of ζ for 3d transition metals. Furthermore, the relation between Δ*E* and ζ is not sufficient to determine the degree of ground-level degeneracy, i.e. the presence of two or more energetically close lying states. Therefore, a more practical criterion is required that identifies not only the existence of ground-level orbital near-degeneracy but also the degree of it.

Herein, we close this gap by a new criterion to identify the presence and degree of ground-level degeneracy from experimentally determined metal-ion magnetic-anisotropy (***g**-* and ZFS-tensors). The starting point of our study was the establishment of a detailed magneto-structural correlation in the three-coordinate Fe complex [(IMes)Fe(dvtms)] (IMes = 1,3-di(2′,4′,6′-trimethylphenyl)imidazol-2-ylidene, dvtms = divinyltetramethyldisiloxane, **1**), stabilized by a ligand set of an NHC and two alkenes.^18^ Accurate magnetic characterization of complex **1** was obtained, by measuring the *g*- and ZFS-values by complementary SQUID magnetometry and broadband Frequency-Domain Fourier-Transform THz-EPR (FD-FT THz-EPR). Quantum chemical calculations established a correlation between the measured spin Hamiltonian (SH) parameters and the electronic structure of **1**. This approach, however, does not allow the individual magneto-structural correlation derived for **1** to be readily transferred to systems with analogous electronic structures. To obtain the desired general criterion, we therefore extended our theoretical approach and introduced an effective Hamiltonian (EH)^8^ coupled with wave-function-based *ab initio* calculations. This EH treats the energy differences between the orbital ground state and the low-lying excited states on the same footing as the SOC and the magnetic interactions between them. Our approach allows generalized statements to be made about the relationship between *g-* and ZFS-values and the degree of near-degeneracy of the orbital ground level. In the following, we distinguish between double and triple near-degeneracy. Triple degeneracy is further subdivided into type I, in which the energy gap between the first and the second excited state is small compared to their energy separation from the ground state, and type II, in which the distance from the ground state to the first excited state is comparable to the distance between the two excited states.

For more than half-filled shells we found that doubly degenerate levels exhibit *g*_⊥_ < 2 < *g*_∥_ and *D* < 0, type I triply degenerate levels have *g*_∥_ < 2 < *g*_⊥_ and *D* > 0, while type II triply degenerate levels have *g*_*x*_ < 2 < *g*_*y*_ < *g*_*z*_ and *D* < 0. Systems having less than half-filled degenerate shells are differentiated by *g*_⊥_ < *g*_∥_ < 2 and *D* > 0 for type I triple degeneracy, *g*_*z*_ < *g*_*y*_ < *g*_*x*_ < 2 and *D* < 0 for type II triple degeneracy, and *g*_∥_ < *g*_⊥_ < 2 and *D* < 0 for double degeneracy.

In the last part of this work, we apply the newly derived criteria to related metal complexes (see Chart 1) and discuss their magneto-structural correlations.

## Experimental Section

### Synthesis

The complex [(IMes)Fe(dvtms)] **(1)** was synthesized according to the reported procedures.^18^ For all experimental work and storage, inert gas conditions or cryogenic temperatures were maintained.

### Magnetometry

Magnetic susceptibility was obtained with a superconducting quantum interference device (SQUID, MPMS-7, Quantum Design; calibrated with a standard palladium reference sample; error <2%) on powder samples of **1** immobilized in *n*-octadecane at an applied field of 1 T. Diamagnetic background arising from *n*-octadecane was subtracted from raw data. Susceptibility data is plotted as χ*T* vs *T* curves. Variable-field variable-temperature (VFVT) magnetization measurements were carried out at 1, 4, and 7 T on the same SQUID magnetometer with the magnetization equidistantly sampled on a 1/*T* temperature scale in the temperature range 2–300 K.

### THz-EPR Spectroscopy

FD-FT THz-EPR was performed at the THz-EPR beamline of the synchrotron user facility BESSY II.^19^ The spectrometer allows for THz-EPR measurements from 0.1 to 175 THz (∼3–5800 cm^–1^), employing a fully evacuated quasi-optical THz beamline, a high-resolution Fourier-transform infrared (FTIR) spectrometer (Bruker IFS 125HR), a superconducting high-field magnet (Oxford Spectromag 4000; *B*_0_ = +11 to −11 T) with variable temperature insert (VTI, *T* = 2–300 K) and a liquid-He-cooled (4.2 K) bolometer detector (IR labs). A detailed description of the spectrometer can be found elsewhere.^20^ Experimental procedures are described in the Supporting Information (SI). FD-FT THz-EPR spectra are shown as magnetic field division spectra (MDS) (Figure S1). FD-FT THz-EPR spectra are shown as magnetic field division spectra (MDS), where a spectrum obtained at an external magnetic field *B*_0_ + 0.5 T is divided by a reference spectrum measured at *B*_0_.

### Calculational Setup

All calculations were performed with the ORCA 5.0 quantum chemical program.^21^ The crystal structure of complex **1** was used in highly correlated wave-function-based *ab initio* calculations. In order to reduce the computational costs, methyl functionalities on the phenyls were substituted by hydrogens, and their positions were optimized by using the TPSSh functional^22^ in combination with the def2-SVP basis set^23^ for all atoms. Noncovalent interactions were accounted for using the D3BJ corrections by Grimme.^24^ For complete active space self-consistent field (CASSCF)^25^ computations, the active space is composed of 8 electrons distributed into orbitals including the five 3d orbitals of the Fe center and the two bonding counterparts of the Fe d_*x*^2^–*y*^2^_ and d_*xy*_ molecular orbitals to account for the strong covalent interaction between the dvtms ligand and the Fe atom. In addition, the second d shell is also included into the active space to account for the double-shell effect.^26^ Quasi-restricted orbitals from DFT calculations were chosen as the initial guess of the active orbitals. On top of the CASSCF wave functions, second-order N-electron valence perturbation theory (NEVPT2)^27^ was employed to capture dynamic correlations. CASSCF/NEVPT2 computations used the def2-SVP basis set for C and H atoms and the def2-TZVP basis set for other atoms along with the def2/JK auxiliary basis set^28^ for the RIJK approximation.^29^

## Results and Discussion

### Determination of the Magnetic Anisotropy in **1**

**Figure 1:**
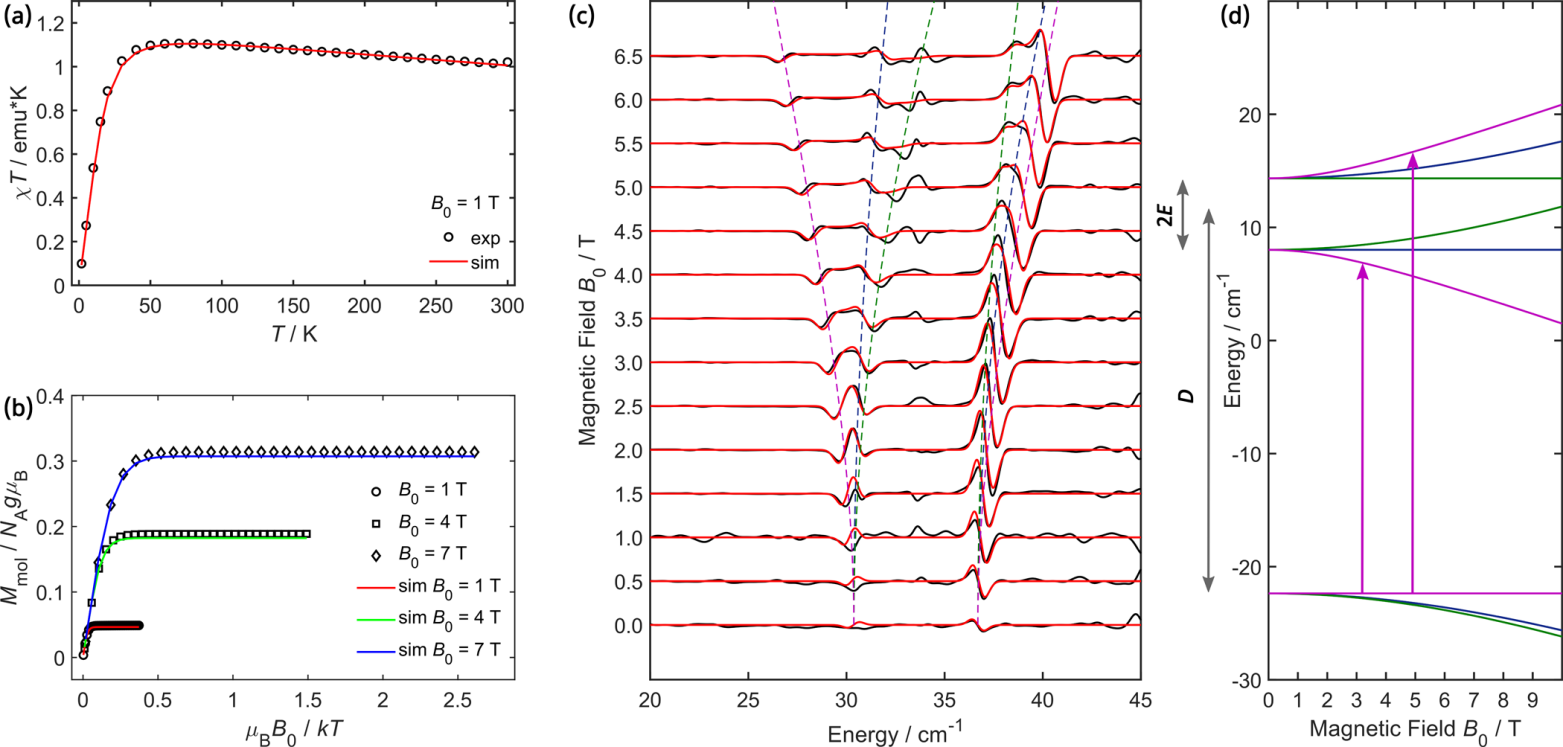
Magnetic characterization of **1**, (a) χ*T* vs *T* at 1 T, (b) VFVT curves obtained at 1, 4 and 7 T and (c) FD-FT THz-EPR MDS at *T* = 4.8 K. Simulations (colored lines) obtained with eq 1 and parameters given in the text and Table S1 are plotted alongside experimental data (circles for χ*T*, circles, squares and diamonds for VFVT data and solid black lines for FD-FT THz-EPR spectra). Dashed lines in (c) indicate simulated field dependent transition energies for ***B***_**0**_ aligned along the *x*- (blue), *y*- (green) and *z*-axes (magenta) of the ZFS-tensor. (d) *S* = 1 spin-energy levels vs external magnetic field calculated with the same SH model and plotted with the same color code as in (c). Vertical arrows indicate allowed EPR-transitions for the *z*-axis projection.

For precise determination of the magnetic anisotropy of **1**, χ*T* and VFVT measurements were complemented with FD-FT THz-EPR spectroscopy (Figure 1a–c, respectively). The χ*T* curve of **1** depicted in Figure 1a exhibits a room temperature value close to 1 emu·K (μ_eff_ = 2.83 μ_B_), the anticipated spin-only value for an *S* = 1 state. Further lowering the temperature below ∼50 K leads to a sharp drop in χ*T*, indicating substantial ZFS. The corresponding VFVT traces at 1, 4, and 7 T are shown in Figure 1b.

For an independent and direct determination of *D* and *E*, FD-FT THz-EPR spectra of **1** were recorded at 4.8 K and at magnetic fields between *B*_0_ = 0 and 7 T in 0.5 T steps. At low magnetic fields, two resonances are observed at 30 and 36 cm^–1^ in the MDS (Figure 1c), which shift and further split upon increasing the magnetic field. The observed resonances are assigned to Δ*M*_*S*_ = ± 1 transitions between the levels of the *S* = 1 system. As can be seen from the energy levels in Figure 1d, the center of the two transitions around ∼33 cm^–1^ corresponds to *D*, the axial ZFS parameter, while their separation of ∼6 cm^–1^ is determined by 2*E*, the rhombic part of the ZFS. The ability to unambiguously resolve *D* and *E* in the EPR spectrum allows for higher accuracy in the determination of the other SH parameters. In particular, for SQUID magnetometry, the rhombicity of the ZFS tensor is usually strongly correlated with the anisotropy of the ***g***-tensor.

Simulations were performed with the following SH model
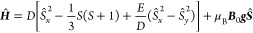
1Here, the first term describes the axial and rhombic second-order ZFS interactions with parameters *D* and *E*, respectively. The coordinate system is molecule-fixed and chosen such that 0 ≤ *E*/*D* ≤ 1/3, with the *z*-axis defining the dominant anisotropy direction. The second term denotes the electron-spin Zeeman interaction involving the ***g***-tensor with the main values, *g*_*x*_, *g*_*y*_ and *g*_*z*_. In the simulations, *g*_*z*_ is assumed as collinear to *D*_*Z*_. THz-EPR spectra and magnetometry data calculated with eq 1 were fitted to the experimental traces by fit routines using the Matlab toolbox EasySpin.^30^ Simulations with the best set of SH parameters are plotted in Figure 1 alongside experimental magnetometry and THz-EPR data. For the THz-EPR data, *D* = +33.54 cm^–1^, *E* = 3.16 cm^–1^ (*E*/*D* = 0.09), *g*_*z*_ = *g*_∥_ = 1.96 and *g*_*x*_ = *g*_*y*_ = *g*_⊥_ = 2.45 were obtained. Note that *g*_∥_, which defines the outer edges of the THz-EPR spectrum, can be determined with higher accuracy than *g*_⊥_. Deviations of the experimental FD-FT THz-EPR spectra from the simulations around 33 cm^–1^ are assigned to spin-phonon couplings,^31^ which are not considered in eq 1.

Since *D* and *E* can be directly read off from the THz-EPR spectra, these parameters were fixed while fitting the SQUID data. For the latter, best agreement between experiment and simulation was obtained for *D* = +33.54 cm^–1^, *E* = 3.16 cm^–1^, *g*_∥_ = 1.97, *g*_⊥_ = 2.23. Overall, excellent agreement between experiment and simulation was achieved. The slight discrepancy between the *g*-values obtained from best fits to the SQUID and THz-EPR data may originate from contributions of the diamagnetic matrix in which complex **1** was fixed during the SQUID experiments. Simultaneous fits of the THz-EPR and SQUID data as described by Lohmiller et al.^32^ yielded nearly the same SH parameters (see Figures S2 and S3 and SH parameters in Table S1). Furthermore, simulations performed using a rhombic ***g***-tensor (*g*_*x*_ ≠ *g*_*y*_ ≠ *g*_*z*_) gave as good results as with an axial ***g***-tensor only when both *g*_*x*_ and *g*_*y*_ were in the range of 2.3 to 2.5. Thus, the following relation of the *g*-values could be unambiguously determined *g*_∥_ = *g*_*z*_ < 2 < *g*_*x*_, *g*_*y*_ or *g*_⊥_. Recently, Neidig and co-workers reported nearly identical *D-* and *E*-values for closely related three-coordinate Fe(0) bis-ethylene complexes, determined with magnetic circular dichroism spectroscopy.^33^ These measurements provide important comparative values for the ZFS- and isotropic *g*-values, but not the degree of *g*-resolution needed to map the full ***g***-tensor anisotropy, as is possible with the FD-FT THz-EPR method used here.

In addition, the sign of *D* could be deduced from analysis of the FD-FT THz-EPR spectra and VFVT curves (Figure S4). For **1**, the energy corresponding to 2*E* (the splitting between the first and the second excited state without magnetic field, Figure 1d) is larger than the thermal energy at the experimental temperature (2*E* = 6.3 cm^–1^ corresponds to ∼9 K > 4.8 K). As the *S* = 1 sublevels are occupied according to Boltzmann statistics, the relative intensities of the Δ*M*_*S*_ = ±1 spin transitions are very sensitive to the sign of *D*. For positive *D* (situation shown in Figure 1), both Δ*M*_*S*_ = ± 1 transitions from the *M*_*S*_ = 0 magnetic ground microstate have equal intensity. For negative *D*, *M*_*S*_ = −1 becomes the energetically lowest lying magnetic microstate, carrying most of the spin population. Therefore, the transition from this level to *M*_*S*_ = 0 would result in much more intense EPR absorption than the transitions between the nearly unoccupied excited magnetic microstates.

Typically, SOC of excited states into the ground state represents the predominant contributions to ZFS in transition-metal complexes. Hence, the observed large positive *D*-value for **1** indicates the presence of low-lying excited states. Furthermore, *g*_⊥_, being considerably larger than *g*_e_, signals the existence of unquenched orbital angular momentum, again arising from SOC with closely spaced excited states. Despite this, as has been shown elsewhere^34^ and verified herein, a pure *S* = 1 SH model suffices to parametrize magnetic properties of a low-lying magnetic triplet even for those having significant unquenched orbital angular momentum. In other words, one can successfully reproduce EPR and magnetometry data using a simple SH (eq 1). Use of a more elaborate Hamiltonian that explicitly accounts for orbital angular momentum and its interactions, such as a Griffith Hamiltonian,^35^ is not necessary.

Despite their usefulness for simulating EPR and SQUID data, SH models have clear limitations as they cannot usually establish a direct relationship between the magnetic properties of a complex and its electronic structure without the detour of quantum chemical calculations. This direct relationship, however, is a necessary prerequisite for a systematic investigation of the nature of the ground-level near-degeneracy via the magnetic anisotropy. To directly relate magnetic properties and electronic structure, a different EH formalism needs to be formulated. To do so, a set of states, a so-called model space, is preselected, and their energy separations are parametrized with a limited number of undetermined factors. Furthermore, one assumes that SOC and electronic Zeeman interactions involving any state out of the model space can be neglected, while exclusively those within the model space need to be considered. Hence, first wave-function-based *ab initio* computations were performed to prescreen excited states that likely make dominant contributions to the magnetic properties of the ground level of **1**. This substantially reduces the number of unknown energy gaps among them and the dimension of the resulting matrix of the EH. In this aspect, it differs from usual ligand field type analyses, which often require a large basis dimension, because of the lack of prescreening, and usually yield a range of interconnected parameters. The complexity of ligand-field models essentially foiled attempts to pinpoint key electronic-structure features that are responsible for unusual magnetic properties.^36^ In contrast, the EH approach is well-suited for rationalizing magnetic interactions of systems featuring orbital near-degeneracy.

### Calculations of the Electronic Structure of **1**

**Figure 2:**
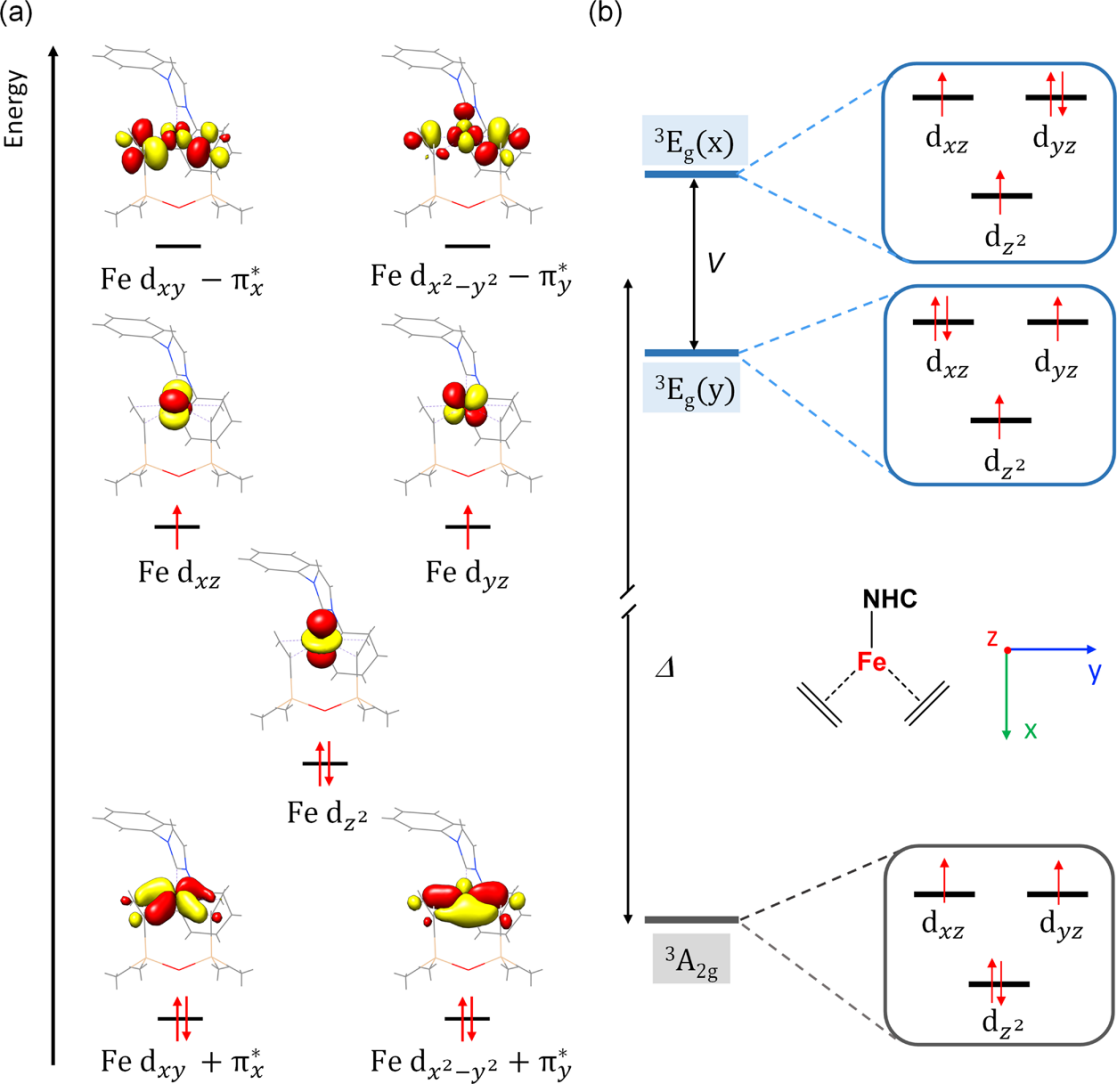
(a) Active orbitals and orbital occupation of the ^3^A_2g_ ground state of **1** obtained by CASSCF calculations. (b) Low-energy spectrum with the near-degenerate orbital set of d_*z*^2^_, d_*xz*_ and d_*yz*_, the resulting ground state ^3^A_2g_ and the two excited states ^3^E_g_(*x*) and ^3^E_g_(*y*) labeled by the irreducible representations of the effective *D*_3_ point group. Δ denotes the energy difference between the ground state and the barycenter of the first and second excited state, and *V* represents the gap between them.

As shown in Figure 2a, CASSCF(8,12)/NEVPT2 computations revealed that the ground state of **1** consists of a leading electron configuration of (d_*x*^2^–*y*^2^_ + π_y_^*^)^2^(d_*xy*_ + π_x_^*^)^2^(d_*z*^2^_)^2^(d_*yz*_)^1^(d_*xz*_)^1^, which accounts for 79% of the wave function. In the present case, due to the considerable π-accepting capability of the C=C moieties of dvtms, the Fe d_*xy*_ and d_*x*^2^–*y*^2^_ atomic orbitals are substantially stabilized by strong π-backdonation to the formally unoccupied C=C π* orbitals, thereby leading to two pairs of bonding and antibonding molecular orbitals denoted d_*xy*_ ± π_x_^*^ and d_*x*^2^–*y*^2^_ ± π_y_^*^. Such interactions are rather covalent as implied by nearly identical percentages of Fe 3d and C=C π* contributions in d_*xy*_ ± π_x_^*^ and d_*x*^2^–*y*^2^_ ± π_y_^*^. While the Fe d_*z*^2^_, d_*xz*_ and d_*yz*_ atomic orbitals are essentially nonbonding in nature, the 3d_*z*^2^_ based orbital gets somewhat stabilized because of mixing with 4s and becomes doubly occupied in the ground state.

*Ab initio* computations further suggest that the first and second excited states of **1** arise from single excitations from d_*z*^2^_ to d_*yz*_ and to d_*xz*_, and thus possess dominant electronic configurations of (d_*x*^2^–*y*^2^_ + π_y_^*^)^2^(d_*xy*_ + π_x_^*^)^2^(d_*z*^2^_)^1^(d_*yz*_)^2^(d_*xz*_)^1^ and (d_*x*^2^–*y*^2^_ + π_y_^*^)^2^(d_*xy*_ + π_x_^*^)^2^(d_*z*^2^_)^1^(d_*yz*_)^1^(d_*xz*_)^2^ (Figure 2b), respectively. The third and fourth excited states are of primary ligand-to-metal charge transfer (LMCT) character, originating from excitations from d_*xy*_ + π_x_^*^ to d_*yz*_ and d_*x*^2^–*y*^2^_ + π_y_^*^ to d_*yz*_. As illustrated in Table S4, irrespective of the number of excited states and the spin multiplicities under investigation, CASSCF(8,12)/NEVPT2 computations invariably deliver a low energy gap (Δ) of less than 2500 cm^–1^ and a marginal splitting *V* ≈ 200 cm^–1^ between the two excited states. The third and fourth excited states were computed to lie more than 16,000 cm^–1^ above the ground state. It should be pointed out that the computed energy gaps among the ground state and the first and second excited states fall into the uncertainty range of wave-function-based *ab initio* quantum chemical calculations and hence may not be quantitatively accurate. Despite this uncertainty, the SH parameters, *D* = 34.7 cm^–1^, *E*/*D* = 0.06 cm^–1^, *g*_*x*_ = 2.45, *g*_*y*_ = 2.40 and *g*_*z*_ = 1.98, predicted by the three-root computations, agree with the experimental values reasonably well, and calculations targeting an increased number of excited states do not discernibly improve the results. Remarkably, irrespective of the number of roots considered, the lowest *g*-value was computed to be invariably smaller than *g*_e_ (Table S4), which is rather unusual for a mononuclear transition metal complex having a more than half-filled d shell.

### Effective Hamiltonian for Complexes with Near-Degenerate Ground Levels

Although CASSCF/NEVPT2 calculations reproduce the SH parameters of **1**, the underlying physical origins remain elusive and more critically, insights deduced from calculations on an individual complex hardly can be carried over to similar systems. To set up a generalized magneto-structural correlation, an EH is derived to explicitly consider the SOC and the electron Zeeman interaction in the basis of the low-lying electronic triplet as suggested by *ab initio* computations (Figure 2b). For *V* = 0, the system has effective *D*_3_ symmetry, and states ^3^A_2g_, ^3^E_g_(*x*) and ^3^E_g_(*y*) can be readily distinguished. To be consistent, even for *V* ≠ 0 the same labels will be used. Therefore, the EH operator, ***Ĥ***_EH_, consists of three terms (eqs 2a–2c)
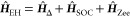
2a
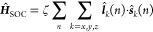
2b
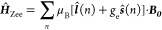
2c***Ĥ***_Δ_ is the unperturbed nonrelativistic energy gap between ^3^A_2g_ and ^3^E_g_(*x*) and ^3^E_g_(*y*) in the Born–Oppenheimer Hamiltonian. ***Ĥ***_SOC_ and ***Ĥ***_Zee_ account for SOC and Zeeman interactions, including spin and orbital Zeeman terms. ζ = 320 cm^–1^ is the effective SOC constant of Fe(0) obtained from earlier calculations.^37^

The EH is constructed in the basis of the low-lying electronic triplet by utilizing their many-electron basis functions in terms of normalized Slater determinants. For *M*_*S*_ = +1, the many-electron basis functions are given by (eqs S1a-i)
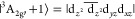
3a
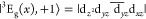
3b
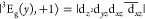
3cA bar (no bar) on top of a given orbital indicates that this orbital is occupied by a spin-down (spin-up) electron. The filled d_*xy*_ + π_x_^*^ and d_*x*^2^–*y*^2^_ + π_y_^*^ orbitals are excluded because the associated excited states, according to the above CASSCF(8,12)/NEVPT2 calculations, lie more than 16,000 cm^–1^ above the ground state. The nonvanishing matrix elements of ***Ĥ***_Δ_ are given by
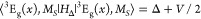
4a
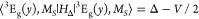
4bShown in Table S2 is the EH matrix in the basis of the nine magnetic sublevels of ^3^A_2g_, ^3^E_g_(*x*) and ^3^E_g_(*y*). To treat the SOC and the electron Zeeman splitting of the magnetic microstates derived from ^3^A_2g_, ^3^E_g_(*x*) and ^3^E_g_(*y*) on the same footing as their energy separations, we did not employ second-order perturbation theory, but diagonalized the EH matrix, which amounts to infinite order perturbation treatments. Thereby, the wave-functions of the three low-lying magnetic levels, |φ_*i*_⟩ (*i* = 1, 2, 3), and their energies at zero field and the variations thereof in the presence of a magnetic field were readily obtained. Furthermore, all SH parameters can be directly extracted. (For details, please refer to the SI). As elaborated in the following, the EH analyses predict *D* = 33.97 cm^–1^, *E* = 3.33 cm^–1^, *g*_*x*_ = 2.423, *g*_*y*_ = 2.509 and *g*_*z*_ = 1.977, alongside Δ = ≈ 1800 cm^–1^ and *V* = ≈300 cm^–1^. As such, the obtained SH parameters are all in very reasonable agreement with experiment, and the energy gaps between the unperturbed electronic triplets are close to those estimated by *ab initio* CASSCF(8,12)/NEVPT2 computations.

**Figure 3:**
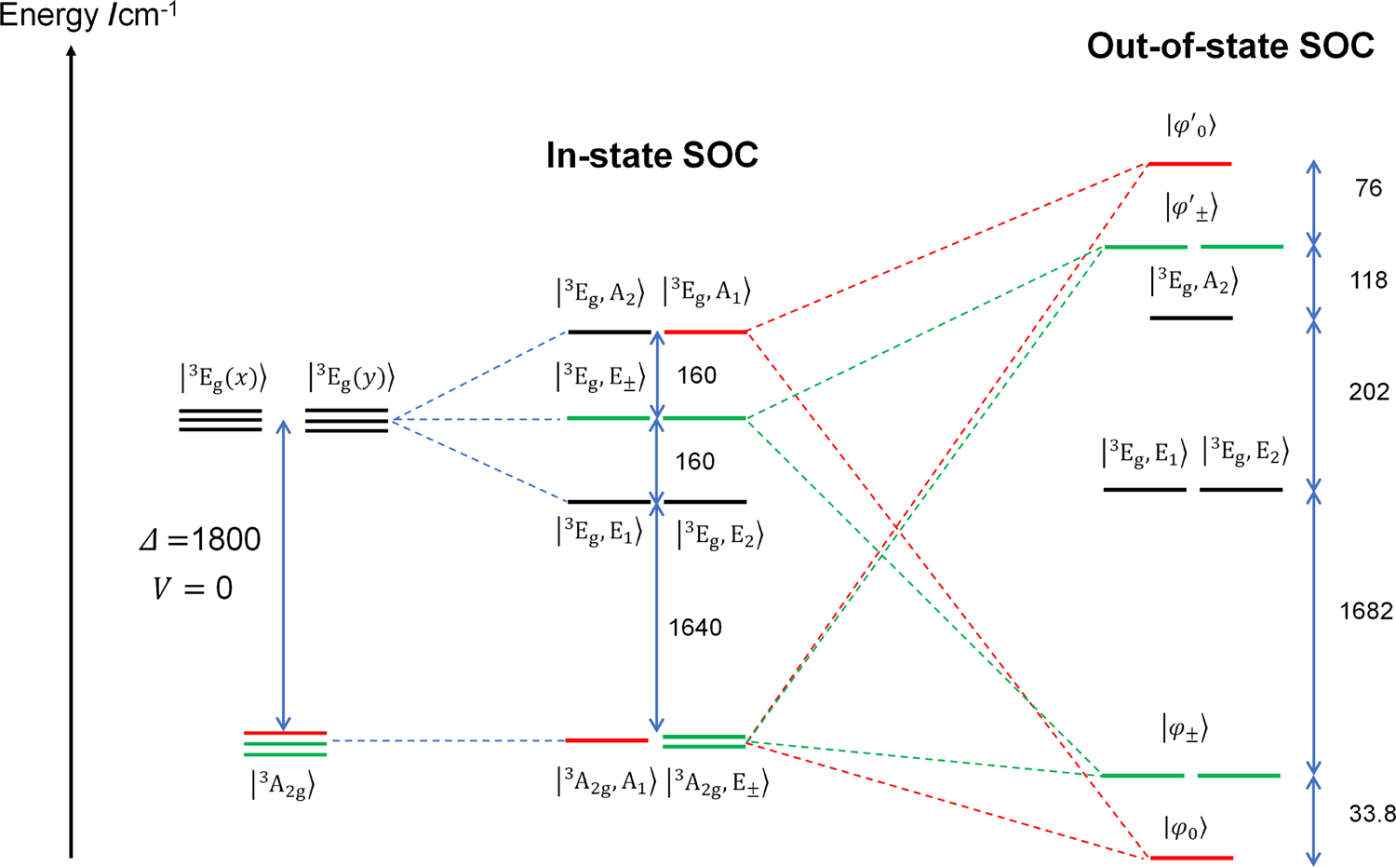
Energy spectra considering no SOC (left), in-state SOC (middle) and out-of-state SOC (right) with ζ = 320 cm^–1^, Δ = 1800 cm^–1^ and *V* = 0 cm^–1^ (see eqs 4a, 4b and Figure 2 for the definition of Δ and *V*). In-state SOC considers only the interactions among components of the same irreducible representation, i.e. the two components of ^3^E_g_, and out-of-state SOC deals with all SOC interactions between ^3^A_2g_ and ^3^E_g_ (eqs 3a–3c). Magnetic microstates resulting from in-state SOC of ^3^E_g_ are labeled |^2*S*+1^Γ_1_, Γ_2_⟩, where Γ_1_ and Γ_2_ are the irreducible representations of *D*_3_ and its double group (see eqs S11–S13 in the SI), respectively. Dotted red and green lines represent the two distinct channels of out-of-state SOC between ^3^A_2g_ and ^3^E_g_, respectively. |φ_0_⟩,|φ_0_^′^⟩,|φ_±_⟩,|φ_±_^′^⟩ (eqs 7a–7c) represent the six magnetic sublevels arising from the out-of-state SOC. Those magnetic sublevels displayed in black are unaffected by the out-of-state SOC.

The SH parameters determined experimentally indicate that complex **1** features a pseudoaxial symmetry as evidenced by a small *E*/*D* value of ∼0.1. To simplify our analyses and gain a better understanding of the physical origin of the magnetic anisotropy measured for **1**, we first assumed effective *D*_3_ symmetry and then considered the effect of orthorhombic distortion *V* afterward. As shown in Figure 3, when ^3^E_g_(*x*) and ^3^E_g_(*y*) are exactly degenerate, as elaborated in our earlier work,^8^ the first-order SOC, so-called in-state SOC, restores orbital angular momentum along the *z-*direction, and splits the ^3^E_g_ state into three accidentally degenerate doublets. The second-order SOC, often referred to as out-of-state SOC between the ^3^A_2g_ and ^3^E_g_ states, lifts the two-fold degeneracy of the first, third and fourth doublets. In the absence of an external magnetic field, diagonalizing the ***H***_**Δ**_ and ***H***_SOC_ parts of the EH matrix yields the wave functions of the nine magnetic sublevels. Of them, the lowest-energy one, denoted |φ_0_⟩, is nondegenerate, and two degenerate microstates, denoted |φ_±_⟩, are the next higher ones in energy (Figure 3).

As such, the situation corresponds to a fictitious *S̃* = 1 spin system (|1,*M*_*S*_⟩, *M*_*S*_ = 0, ± 1) with easy-plane magnetization in the SH formalism, and |φ_0_⟩ and |φ_±_⟩ are hence equivalent to |1,0⟩ and |1, ±1⟩, respectively. Therefore, the axial ZFS parameter, *D*, is simply the energy difference between |φ_0_⟩ and |φ_±_⟩
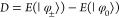
5Analogously, the *g*-values can be computed as follows^8,34^
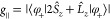
6a
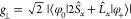
6bFigure 4 shows the SH parameters, *D*, *g*_∥_ and *g*_⊥_ computed as a function of Δ. At Δ = 5.6ζ = 1800 cm^–1^, the best match between the calculated and the experimental *D*-values was achieved. With this Δ-value, we examined the state composition of the nine magnetic sublevels and dissected the physical origins of the magnetic anisotropy. Because out-of-state SOC admixes ^3^E_g_ into ^3^A_2g_, the wave functions of the final low-lying magnetic triplet are given by the following expressions:
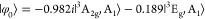
7a
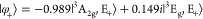
7b
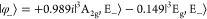
7c

**Figure 4:**
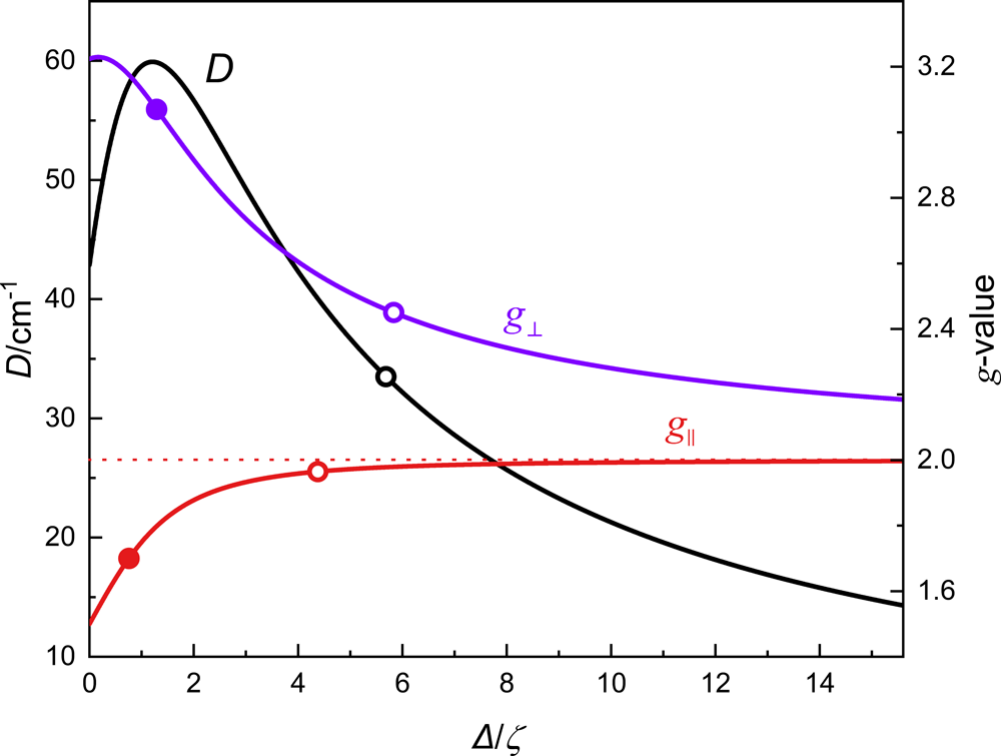
Calculated *D* (black), *g*_∥_ (red) and *g*_⊥_ (purple) plotted vs Δ/ζ (see eqs 5, 6a and 6b, respectively). The open circles indicate the experimental SH parameters for complex **1** determined from THz-EPR (Table S1), while the closed circles represent the experimental *g*-values of **3** reported in ref (8).

Therefore, |φ_0_⟩, and |φ_±_⟩ contain 4% and 2% contribution of |^3^E_g_, A_1_⟩ and |^3^E_g_, E_±_⟩, respectively (compare Figure 3), and those minor, yet discernible parentages of ^3^E_g_ in the low-lying magnetic triplet suggest that the symmetrized complex **1** features a nearly triply degenerate ground level.

**Figure 5:**
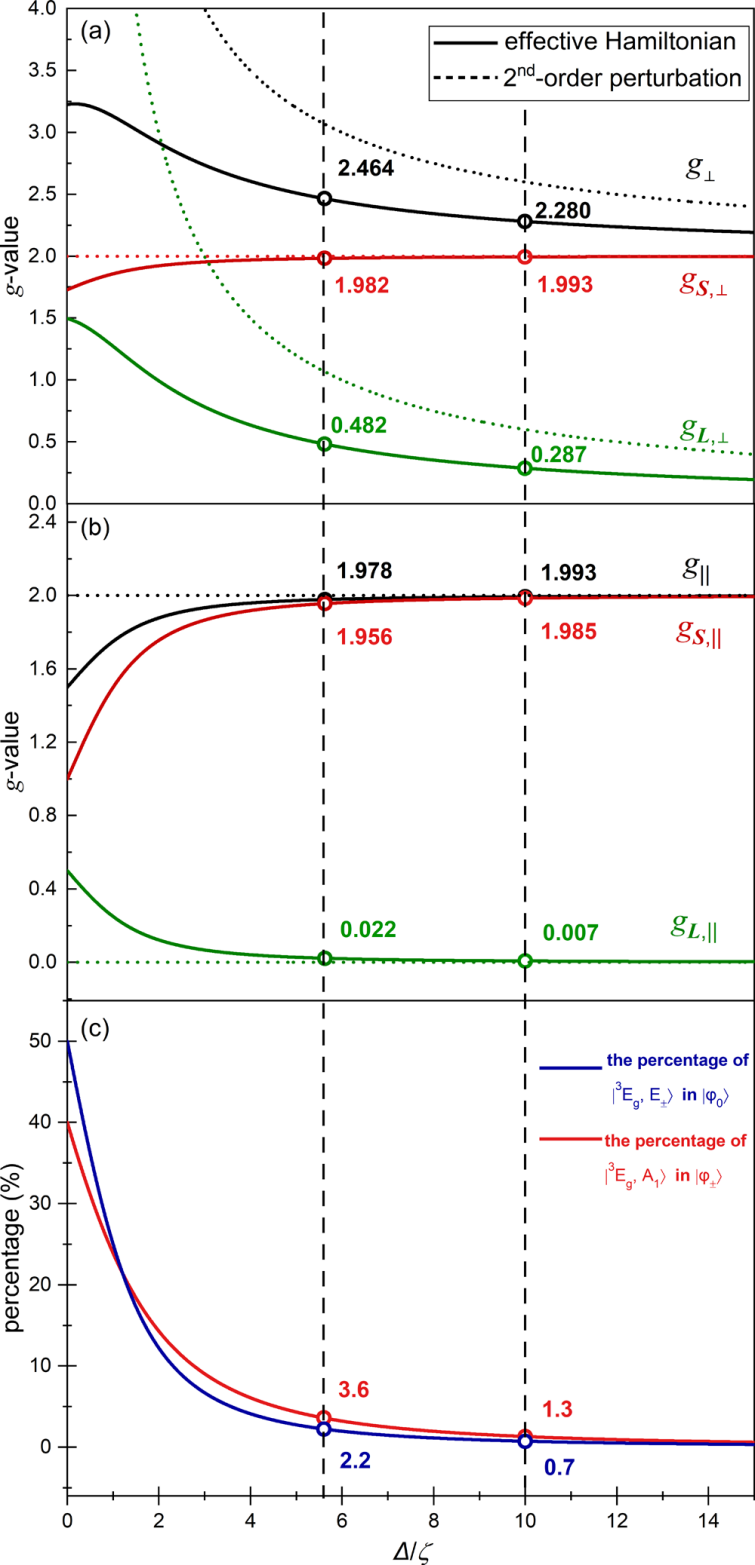
Variation of (a) *g*_*S*,⊥_, *g*_*L*,⊥_, *g*_⊥_, (eqs 6b, 8c and 8d, respectively) (b) *g*_*S*,∥_, *g*_*L*,∥_, *g*_∥_, (eqs 6a, 8a and 8b, respectively) and (c) the respective percentages of |^3^E_g_, E_±_⟩ in |φ_0_⟩ and |^3^E_g_, A_1_⟩ in |φ_±_⟩ as a function of Δ/ζ, derived from EH analysis (solid lines). Dotted lines represent *g*-values computed by second-order perturbation theory. Dashed vertical lines indicate the corresponding values calculated for complex **1** with Δ = 5.6ζ = 1800 cm^–1^ and for systems with Δ/ζ = 10, respectively.

Figure 5 depicts the spin (*g*_*S*,∥_ and *g*_*S*,⊥_) and orbital (*g*_*L*,∥_ and *g*_*L*,⊥_) contributions to *g*_∥_ and *g*_⊥_ as a function of Δ derived from EH analyses (solid lines) and second order perturbation theory (dotted lines), respectively. Apparently, *g*_∥_ and *g*_⊥_ estimated by second-order perturbation theory exceed the values calculated by EH analysis. Second-order perturbation theory predicts that a single-electron excitation from a doubly to a singly occupied orbital, like in the case of **1** (see Figure S10), leads to *g*-values equal or larger than *g*_e_. This contrasts our experimental findings, which are in line with the predictions from the EH analysis (see Figure 4).
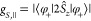
8a
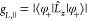
8b
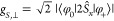
8c
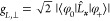
8d

As can be seen from Figure 5, *g*_*L*,⊥_ rises dramatically with decreasing Δ, which originates from the SOC of ^3^E_g_ and ^3^A_2g_, introducing substantial unquenched transverse orbital angular momentum into the low-lying magnetic triplet. For the same reason, *g*_*L*,∥_ completely arises from the orbital angular momentum of the minor contribution of |^3^E_g_, E_+_⟩ to |φ_+_⟩, a third-order term in the perturbative treatment of *g*-shifts.

Unexpectedly, the EH analyses reveal that in the presence of strong SOC both *g*_*S*,∥_ and *g*_*S*,⊥_ are considerably lower than *g*_e_, indicating that SOC can quench the spin angular momentum in all directions. In the present case, *g*_*S*,∥_ entirely stems from the spin angular momentum of the leading component |^3^A_2g_, E_+_⟩ in |φ_+_⟩, because, as elaborated in SI eqs S11e–S12g, |^3^E_g_, E_+_⟩ has a lower eigenvalue of 0 with respect to *Ŝ*_*z*_, viz. ⟨*Ŝ*_*z*_⟩ = 0, than |^3^A_2g_, E_+_⟩, which is distinguished by ⟨*Ŝ*_*z*_⟩ = 1. Therefore, the SOC-induced state admixing of |^3^E_g_, E_+_⟩ into |^3^A_2g_, E_+_⟩ dilutes the spin angular momentum of the latter. The same dilution effect also accounts for *g*_*S*,⊥_ being lower than *g*_e_. Furthermore, |^3^A_2g_, E_+_⟩ and |^3^E_g_, E_+_⟩ are the eigenfunctions of *L̂*_*z*_ with eigenvalues of 0 and +1, respectively, but since *g*_e_ of the spin angular momentum is just twice of that of the orbital angular momentum (which is 1), the decrement of the spin contribution to *g*_∥_ due to the mixing of |^3^E_g_, E_+_⟩ in |φ_+_⟩ exceeds its orbital contribution. As such, *g*_∥_ is less than *g*_e_. Finally, as Δ/ζ decreases from 15 to 0, the weight of |^3^E_g_, E_+_⟩ in |φ_+_⟩ increases, and *g*_∥_ steadily declines from 2 to 1.5. On the contrary, *g*_⊥_ is always significantly larger than *g*_e_, because *g*_*L*,⊥_ is invariably in excess of the difference of *g*_*S*,⊥_ relative to *g*_e_.

As depicted in Figure 5c, when Δ/ζ > 10, the mixing between |^3^E_g_⟩ and |^3^A_2g_⟩ caused by SOC is negligible with minor percentages of |^3^E_g_, E_±_⟩ and |^3^E_g_, A_1_⟩ in |φ_0_⟩ and |φ_±_⟩ being 1.3 and 0.7%, respectively, and so is the quenching of the spin angular momentum of |^3^A_2g_⟩; therefore, 2 = *g*_∥_ < *g*_⊥_, congruent with the *g*-value pattern predicted by perturbation theory. Therefore, Δ/ζ = 10 can be viewed as the turning point where the perturbation treatment on the low-lying electronic triplet breaks down. Because *g*_∥_ is essentially 2 for Δ/ζ in the range of 8–12, the choice of Δ/ζ = 10 as the cutoff to differentiate between systems having nondegenerate or near-degenerate ground levels is somehow arbitrary. But in any case, as the energy splitting of the near-degenerate states declines, the SOC-induced quenching of the spin angular momentum gets increasingly pronounced and hence should be regarded as a more appropriate hallmark to identify whether systems have near-degenerate ground levels or not. Following this line of reasoning, complexes with nearly triple or double degenerate ground levels ought to be distinguished by one or two *g-*values, respectively, smaller than *g*_e_ in the directions where orbital angular momentum is almost quenched.

**Figure 6:**
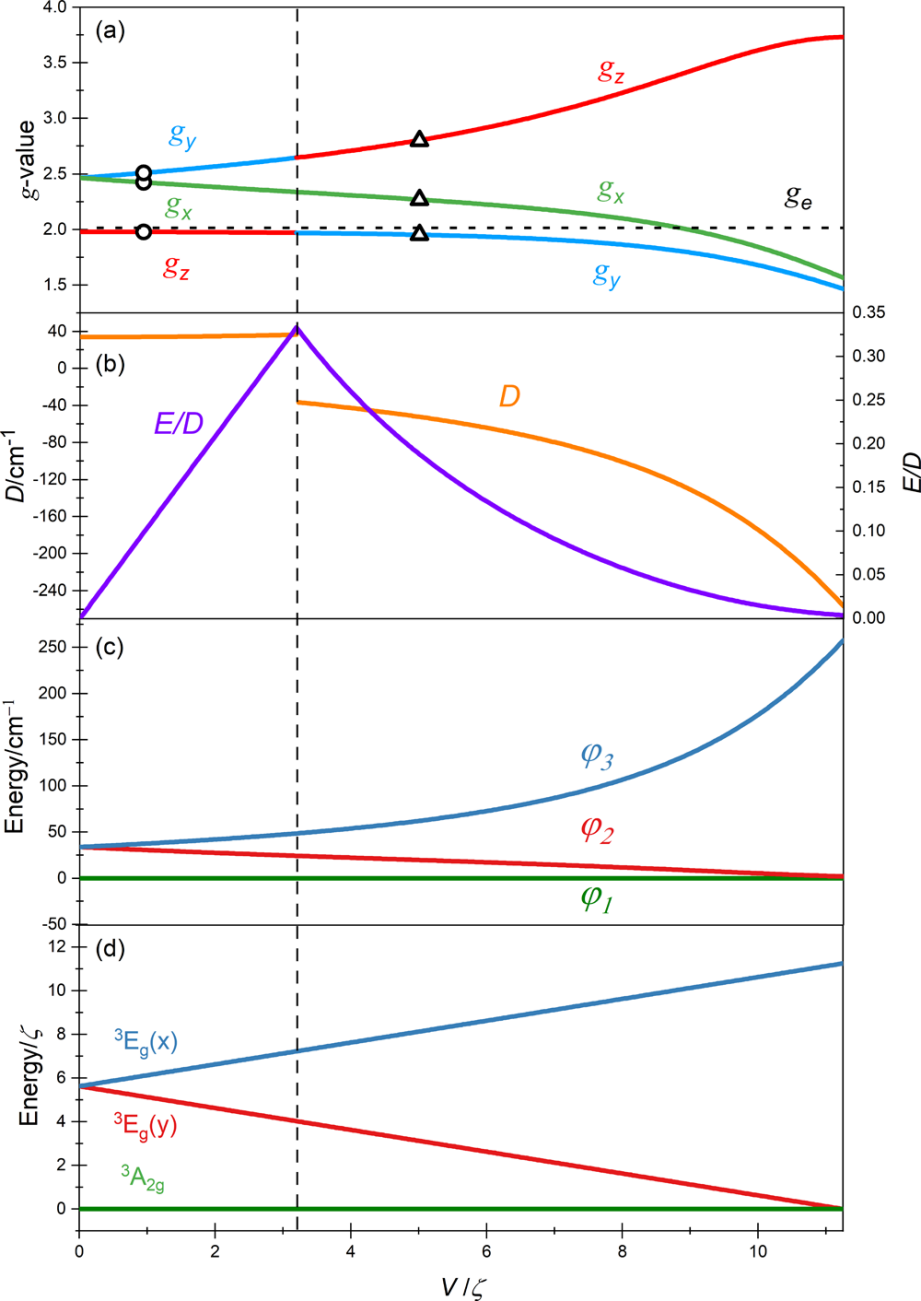
*V*/ζ, dependence of (a) *g*_*x*_ (green), *g*_*y*_ (blue) and *g*_*z*_ (red), (b) the ZFS-values *D* (orange) and *E*/*D* (purple), (c) the three low-lying microstates |φ_*i*_⟩ (*i* = 1, 2 and 3) (green, red and blue) (see eqs S2a–c and Table S2) and (d) the low-lying electronic triplets (see Figure 2b) ^3^A_2g_ (green), ^3^E_g_(*x*) (blue) and ^3^E_g_(*y*) (red). All parameters were calculated for fixed Δ = 5.6ζ = 1800 cm^–1^ with ζ = 320 cm^–1^ (see Figure S7 for further calculations with varying Δ). The black open circles and triangles represent the situations for **1** with *V* = 0.94ζ = 300 cm^–1^ and for complex **6** with *V* = 5ζ = 2450 cm^–1^, respectively. The black dashed line indicates the situation where the three lowest states |φ_*i*_⟩ have equal energy spacing and *E*/*D* = 1/3.

We are now in a position to examine the influence of the rhombic splitting *V* between ^3^E_g_(*x*) and ^3^E_g_(*y*) on the magnetic properties of **1**. Starting from *V* = 0 in Figure 6, where *g*_*z*_ = *g*_∥_ < 2 < *g*_⊥_= *g*_*x*_ = *g*_*y*_, with increasing *V*, *g*_*x*_ declines and *g*_*y*_ rises to *g*_*z*_ < 2 < *g*_*x*_ < *g*_*y*_, which mirrors the energy variations of ^3^E_g_(*x*) and ^3^E_g_(*y*). While increasing *V* from 0, the *D*-value is essentially unchanged, but *E*/*D* increases sharply. Remarkably, at *V* = 3.19ζ = 1020 cm^–1^ (black dashed line in Figure 6), the sign of *D* switches from positive to negative, while *E*/*D* reaches the maximum value of 1/3. When *E*/*D* is close to 1/3, the two energy gaps of the three low-lying magnetic sublevels are nearly identical and one can map the lowest-energy one, |φ_0_⟩, either to *M*_*S*_ = 0 or to *M*_*S*_ = −1. As a result, when *V* > 3.19ζ, the sign of *D* changes, and the principal *z*-axis of the traceless ***D***-tensor transitions from the molecular *z*-axis to the *y-*axis. In experiments, the principal axes of the ***g***-tensor are typically referenced to those of the ***D***-tensor, the three *g*-values are thus relabeled accordingly as *g*_*y*_ < 2 < *g*_*x*_ ≪ *g*_*z*_ in Figure 6. As *V* further increases, the energy gap between ^3^A_2g_ and ^3^E_g_(*x*) substantially exceeds that between ^3^A_2g_ and ^3^E_g_(*y*); consequently, *g*_*x*_ changes from being higher than *g*_e_ to being lower than *g*_e_, resulting in a *g*-value pattern of *g*_*x*_ ≈ *g*_*y*_ < 2 ≪ *g*_*z*_. At the same time, the magnitude of the negative *D*-value constantly ascends, while *E*/*D* reduces. Ultimately, the system is best formulated as having two-fold pseudodegeneracy, as manifested by *D* ≪ 0 and *g*_*x*_*≈ g*_*y*_ = *g*_⊥_ < 2 ≪ *g*_*z*_ = *g*_∥_, even as the energy separations within the low-lying electronic triplet remain below 10ζ. Accordingly, for a (d_*z*^2^_d_*xz*_d_*yz*_)^4^*S* = 1 system with three orbital states lying in an energy range of less than 10ζ, three different scenarios with characteristic magnetic anisotropies can be envisioned (see discussion above and Figure S10).

**Figure 7:**
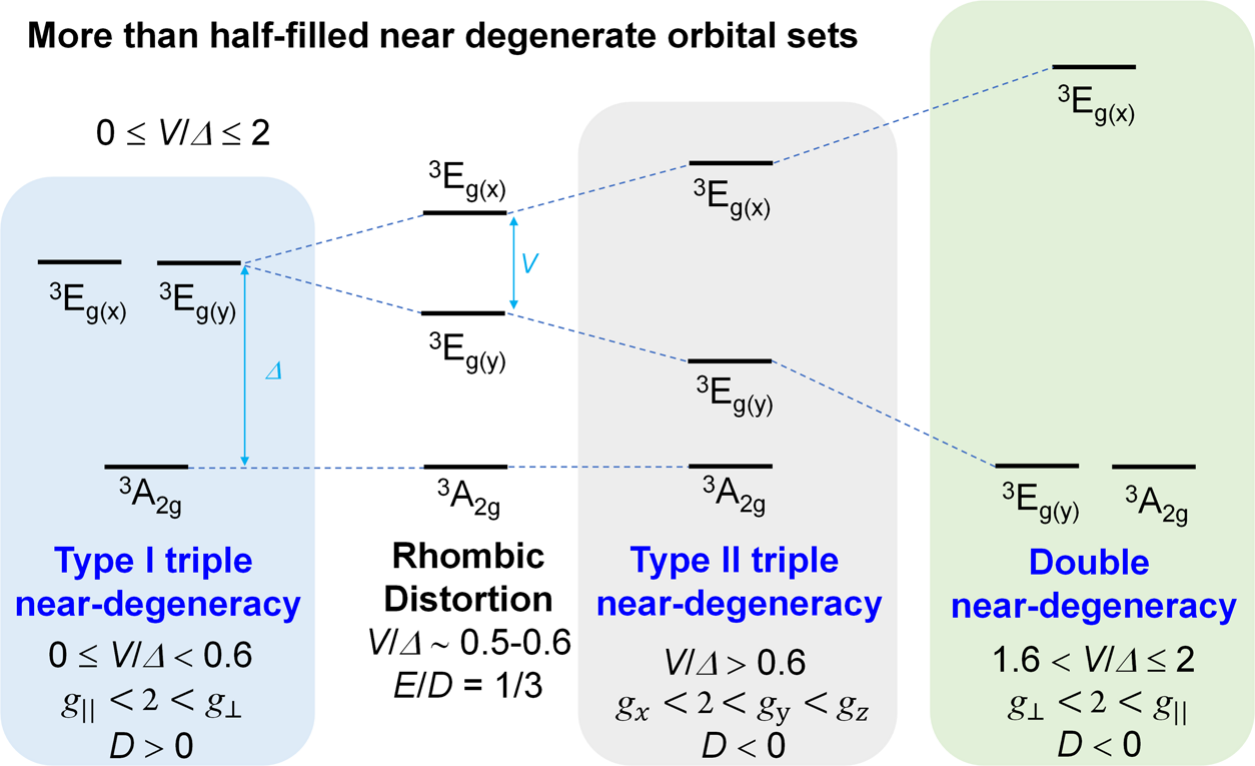
Electronic-structure scenarios for a (d_*z*^2^_d_*xz*_d_*yz*_)^4^*S* = 1 system having three low-lying orbital states. Type I and II triple degeneracy are separated by the configuration where the ZFS tensor reaches its maximum rhombicity.

As shown in Figure 7, first, type I triple near-degeneracy, where the energy gap of the upper two states is negligible compared to their energy difference with respect to the lower state. Second, type II triple near-degenerate levels, with considerable rhombic splitting lifting the quasi-degeneracy of the upper two states and resulting in three nearly equally spaced energy states. Finally, doubly near-degenerate levels have two very close lying states and a third energetically much higher lying state.

To generalize this classification, we explored more situations varying Δ from ζ to 9ζ. As shown in Figure S7, the transition point between type I and II triple near-degeneracy, distinguished to be *E*/*D* = 1/3, is in a narrow range from *V/*Δ = 0.5 to 0.6 upon increasing Δ. However, the boundary between type II triple near-degeneracy and double near-degeneracy depends heavily on Δ. For Δ < 1.25ζ, *V*/Δ < 2 does not allow the existence of double degeneracy. More important is that in the double degenerate regime, the system invariably exhibits the characteristic EPR signature of *g*_*z*_ > 2 > *g*_*x*,*y*_ and *D* ≪ 0, even if the second orbital excited state is situated by less than 10ζ in energy relative to the ground one. As such, one can determine whether one or two excited states dominate the SOC interaction, even for cases where more than three low-lying excited states exist.

### Comparison to other Low-Coordinate Complexes

In the following, magneto-structural correlations derived for complex **1** are compared to related three-coordinate complexes shown in Chart 1. Complexes **4** and **4**′ have *D* = +16.8 cm^–1^ and *E*/*D* = 0.2 and *D* = +14.3 cm^–1^ and *E*/*D* = 0.26, respectively. On the contrary, complex **5** exhibits *D* = −20 cm^–1^ and *E*/*D* = 0.2, despite sharing an analogous geometric structure to **4** and **4**′. Based on the following detailed ligand field analysis of their electronic structures, we reasoned that these three complexes may all possess near-degenerate ground levels as reflected by their appreciable *D*-values, and specifically be in the intermediate region between type I and II triple near-degeneracy as suggested by their sizable *E*/*D*-values (see black dashed line in Figure 6).

**Figure 8:**
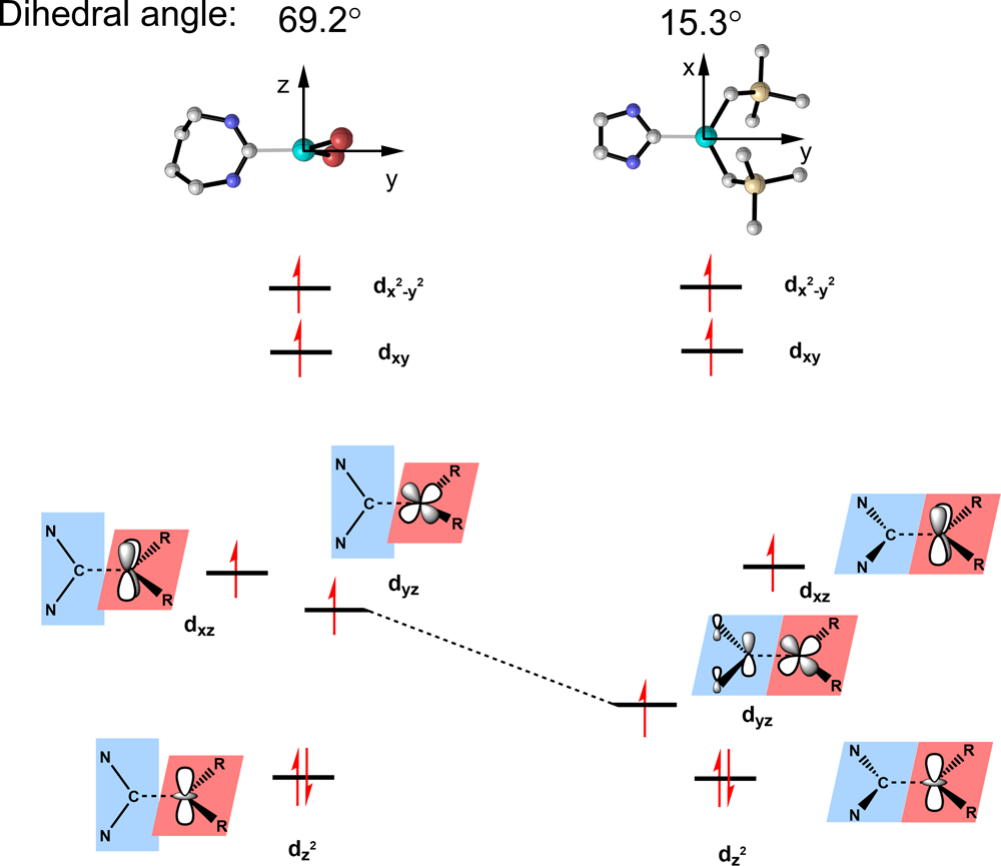
Geometric and electronic structure of **4** and **5**. The dihedral angles formed between the R-Fe-R (R = Br^–^ and CH_2_TMS^–^) plane and the plane of the carbene N–C–N-plane are 65.4 and 15.3° in complexes **4** and **5**, respectively. All carbene substituents are omitted for clarity.

In analogy to the bonding situation of **1**, the strong σ donation of the supporting ligands in **4**, **4**′ and **5** raises the d_*x*^2^–*y*^2^_ and d_*xy*_ orbitals to higher energies, and the remaining d_*z*^2^_, d_*yz*_ and d_*xz*_ orbitals are nearly nonbonding (Figure 8). However, in the case of complex **5**, the d_*yz*_ orbital gets somewhat stabilized, because of its π back-bonding interaction with the NHC π* orbital. Consequently, the energy separation between the d_*z*^2^_ and d_*yz*_ orbitals shrinks, and complex **5** is best interpreted as possessing a type II triply degenerate ground level, in accordance with its negative *D*. In contrast, for complexes **4** and **4**′, the corresponding π back-bonding interaction is much weaker, because of an unfavorable geometry with a much larger dihedral angle of 69.2 and 86.8°, respectively, between the R-Fe-R (R = Br^–^) plane and the carbene N–C–N plane. Therefore, the ground levels of complexes **4** and **4**′ still feature type I triple near-degeneracy consistent with their positive *D*-values.

CASSCF(6,10)/NEVPT2 calculations substantiate the views deduced from the ligand field analysis. As shown in Table S5 and Figures S8 and S9, the greatest energy separations within the low-lying electronic triplets of complexes **4** and **5** were predicted to be less than 2400 cm^–1^, indicating that both species possess near-degenerate ground levels. Specifically, the ground level of **4** features type I triple degeneracy as suggested by *V*/Δ = 0.65, consistent with its positive *D*-value. In the case of **5**, because of the π back-bonding interaction between the NHC π* and Fe d_*yz*_ orbitals, the second excited state has a much lower energy than the third one. Consequently, complex **5** has a type II triply near-degenerate ground level with *V*/Δ = 1.3, congruent with its negative *D*-value and reduced rhombicity as compared to **4**.

Recently, some of us reported that an *S* = 1 three-coordinate Fe(IV) bisimido complex [(IPr)Fe^IV^(NC(CF_3_)_2_Ph)_2_] (**6**)^38^ has an orbitally nearly doubly degenerate ground level. This electronic structure description is congruent with its strong eas*y*-axis magnetization with *D* = −79.2 cm^–1^, *E*/*D* = 0.09, *g*_∥_ ≈ 2.80 and *g*_⊥_ = 1.83, revealed by SQUID magnetometry and applied field Mössbauer spectroscopy. Furthermore, wave-function-based *ab initio* calculations predicted that the dominant ground electron configuration (d_*z*^2^_)^2^(d_*yz*_)^1^(d_*xz*_)^1^ is only 1020 cm^–1^ lower in energy than the first excited state (d_*z*^2^_)^1^(d_*yz*_)^2^(d_*xz*_)^1^. The bonding situation of complex **6** is thus analogous to that of **1** except that the two spectator orbitals, d_*x*^2^-*y*^2^_ and d_*xy*_ are empty in the former. The EH model is therefore applicable to **6** by setting the following parameters to match the largest *g*-value determined experimentally (open triangles in Figure 6a): the effective one-electron SOC constant of Fe(IV) ζ = 487 cm^–1^, Δ = 5.6ζ, *V*/Δ = 0.89. The resulting EH gives *D* = −81.0 cm^–1^, *E*/*D* = 0.18 and *g*_*z*_ = 2.81, *g*_*x*_ = 2.26 and *g*_*y*_ = 1.95. Given the fact that *g*_*x*,*y*_ cannot be accurately determined by experimental spectroscopic characterizations, all values are in reasonable agreement with the experiment, although the simple EH model makes crude approximations, such as neglecting the metal–ligand covalency.

Early work by Holland, Münck, Bominaar and co-worker delineates an electron structure analysis of a series of high-spin (*S* = 2) three-coordinate Fe(II) complexes [LFe^II^Cl] (**2**′, L = β-diketiminate) and [LFe^II^CH_3_] (**2**) with applied-field Mössbauer and parallel-mode EPR spectroscopies.^39^ It has been concluded that they possess orbitally almost doubly degenerate ground levels in concert with their large negative *D*-values (|*D*| > 50 cm^–1^) and unusual effective *g′*_*z*_ = 10.9 (**2**) and *g*′_*z*_ = 11.4 (**2**′) along the *z*-direction of the ***D***-tensor. The effective *g*′-values are obtained by mapping the lowest-energy *M*_*S*_ = ± 2 pseudodoublet of an *S* = 2 multiplet to *M*_*S*_ = ± 1/2 of an effective *S* = 1/2 doublet. Hence, the intrinsic *g*_*z*_-values are 2.73 for **2** and 2.85 for **2**′ because of the vanishing rhombicity, *E/D* ≈ 0.

Very recently, Telser, Holland and co-workers provided more accurate *D*-values for **2** (−38.1 cm^–1^) and **2**′ (−36.9 cm^–1^) measured by frequency-domain far-infrared magnetic resonance spectroscopy (FIRMS) and confirmed their strong eas*y*-axis magnetic anisotropy.^40^ By contrast, as to the *S* = 3/2 Co^II^ congeners, [LCo^II^Cl] (**7**) was found to feature strong easy-plane magnetic anisotropy as manifested by *D* = +55 cm^–1^, alongside effective *g*′_*x*_ = 4.60, *g*′_*y*_ = 5.64 and *g*′_*z*_ = 1.97, whereas [LCo^II^CH_3_] (**7**′) was characterized as having *D* = −49 cm^–1^ and *g*′_*z*_ = 8.50. For complexes **7** and **7**′, *E*/*D* are very low and cannot be precisely determined. If *E*/*D* is assumed to be zero, then the effective *g*′-values can be converted to the intrinsic *g*-values as follows, *g*_⊥_ ≈ 2.56 and *g*_∥_ = 1.97 for **7** and *g*_∥_ ≈ 2.83 for **7**′.

*Ab initio* calculations revealed that three low-lying excited states exist above the ground state within an energy gap of 2000 cm^–1^, arising from closely spaced d_*z*^2^_, d_*xz*_ and d_*yz*_ orbitals.^40^ Despite this complexity, **7**′ was computed to possess a doubly near-degenerate ground level, consistent with the measured *D-* and *g*-values, while **7** has type I triple near-degeneracy, also in line with the EPR findings. The difference can be traced back to the fact that a methyl group is a pure σ donor, but chloride is a σ and π donor. This disparate property of the axial ligands changes the energy of d_*yz*_ relative to d_*z*^2^_ and causes the varying degrees of orbital near-degeneracy as proposed by Telser, Holland and co-workers (see the Supporting Information in ref (40)). These two examples support our conclusion that the *g*-anisotropy and the sign of *D* can be used to determine the number of lowest-energy orbital excited states that strongly interact with the ground state via SOC even for those difficult cases where a range of low-lying excited states exist.

### General Criteria to Identify the Degree of Ground-Level Near-Degeneracy from *g*- and ZFS-Values

Up to here, we have established a magneto-structural correlation for three-coordinate systems. In the following, we generalize our above EH analysis and propose criteria to identify the degree of ground-level near-degeneracy (Table 1). For complexes having more than half-filled degenerate shells, the spin and orbital angular momenta in the low-lying magnetic sublevels orient roughly along the same direction as implied by Hund’s third rule, which provides a rationale for the *g*-shift predicted by second-order perturbation theory (see discussion above and Figure S10). As a consequence, the *g*-value along the direction of the SOC interaction should be larger than *g*_e_. Whereas due to SOC-induced state mixing, the other *g*-components should be less than *g*_e_. Hence, type I triply and doubly near-degenerate systems feature distinct ***g***-tensor anisotropy, namely *g*_⊥_ < 2 < *g*_∥_ (*g*_*x*,*y*_ < 2 < *g*_*z*_) and *g*_∥_ < 2 < *g*_⊥_ (*g*_*z*_ < 2 < *g*_*x*,*y*_), respectively. In between these two limiting situations, type II triply near-degenerate systems are likely distinguished by *g*_*x*_ < 2 < *g*_*y*_ < *g*_*z*_. By contrast, in the case of complexes having less than half-filled degenerate orbitals, the spin and orbital angular momenta in the low-lying magnetic sublevels align approximately along the opposite direction. Therefore, ***g***-tensor anisotropies of *g*_⊥_ < *g*_∥_ < 2 (*g*_*x*,*y*_ < *g*_*z*_ < 2), *g*_*z*_ < *g*_*y*_ < *g*_*x*_ < 2 and *g*_∥_ < *g*_⊥_ < 2 (*g*_*z*_ < *g*_*x*,*y*_ < 2) can be regarded as spectroscopic markers for type I and type II triply, and doubly near-degenerate systems, respectively. Furthermore, systematic EH analyses validated by experimental findings reveal that type I triply near-degenerate systems invariably feature positive *D*-values, whereas type II triply and doubly degenerate systems exhibit negative *D*-values. As classical examples of the latter situation, the (quasi-)linear coordination environment of Fe(I/II)^12^ and Co(I/II)^13^ complexes renders 3d orbitals of π or δ symmetry essentially nonbonding in nature; consequently, these systems were unanimously found to feature strong eas*y*-axis magnetic anisotropy.

**Table 1:** Criteria for the Assignment of Type I and II Triply and Doubly Near-Degenerate Ground States by Their Magnetic Anisotropy

	more than half-filled orbital sets	less than half-filled orbital sets
type I triple degeneracy	*g*_∥_ < 2 < *g*_⊥_, *D* > 0	*g*_⊥_ < *g*_∥_ < 2 *D,* > 0
type II triple degeneracy	*g*_*x*_ < 2 < *g*_*y*_ < *g*_*z*_, *D* < 0	*g*_*z*_ < *g*_*y*_ < *g*_*x*_ < 2, *D* < 0
double degeneracy	*g*_⊥_ < 2 < *g*_∥_, *D* < 0	*g*_∥_ < *g*_⊥_ < 0, *D* < 0

For systems having less than half-filled degenerate orbital sets and featuring two-fold pseudodegeneracy, *S* = 1/2 tetragonal iron(V)-nitrido complexes serve as good examples. Independent of their supporting ligands, these species are invariably distinguished by *g*_∥_ ≈ 1 and *g*_⊥_ ≈ 1.73, because their sole unpaired electron populates doubly near-degenerate Fe–N π* orbitals and their ground levels possess two-fold near-degeneracy.^41^ Very recently, some of us reported EPR characterizations of monosubstituted Sn(I) and Pb(I) doublet radicals. The same ***g***-tensor patterns with ***g*** = [1.957, 1.896, 1.578] for Sn(I) and ***g*** = [1.496, 1.166, 0.683] for Pb(I) were detected.^42^ The observed ***g***-anisotropies are a consequence of their nearly doubly degenerate ground levels with an unpaired electron distributed between almost degenerate nonbonding valence p_*x*_ and p_*y*_ orbitals.

For systems having more than half-filled degenerate orbital sets and featuring type I triple degeneracy, previous work of ours^8^ unequivocally established that the square-planar *S* = 1 Fe(II) porphyrin complex [Fe^II^(TPP)] (**3**) possesses a predominant electron configuration of (d_*xy*_)^2^(d_*z*^2^_)^2^(d_*xz*_)^1^(d_*yz*_)^1^, which lies slightly below (d_*xy*_)^2^(d_*z*^2^_)^1^(d_*xz*/*yz*_)^3^ by 950 cm^–1^ because in square-planar coordination arrangements, d_*z*^2^_, d_*xz*_ and d_*yz*_ are all essentially nonbonding in nature and hence almost energetically degenerate. This peculiar electronic structure is manifested by *g*_∥_ = 1.70, *g*_⊥_ = 3.07 and *D* = +94 cm^–1^ (filled circles in Figure 4) and key to its superior electrochemical activity toward CO_2_ catalytic reduction. The experimental *D*-value of **3** exceeds that predicted by the EH for complex **1**, simply because *D* is not proportional to ζ but to ζ^2^. Similarly, its *S* = 1/2 Co(II) analog, [Co^II^(*p*-OCH_3_)(TPP)] (**8**), also features a type I triply degenerate ground level as implied by *g*_∥_ = 1.80 and *g*_⊥_ = 3.32.^43^ For **8**, the lowest-energy electron configuration was found to be (d_*xy*_)^2^(d_*xz*/*yz*_)^4^(d_*z*^2^_)^1^, which is close in energy to the doubly degenerate (d_*xy*_)^2^(d_*xz*/*yz*_)^3^(d_*z*^2^_)^2^. The same holds true for [Rh^II^(TMP)] (**9**, TMP = meso-tetramesitylporphyrin) with *g*_∥_ = 1.92 and *g*_⊥_ = 2.65. Raising the energy of the d_*z*^2^_ orbital in **8** and **9** by axial coordination with pyridine, CO and other ligands lifts the three-fold near-degeneracy and renders *g*_⊥_ steadily approaching 2.0, depending on the σ-donating strength of the ligand.^44^ For example, the five-coordinate pyridine and CO adducts of **8** were distinguished by *g*_∥_ = 2.02 and *g*_⊥_ = 2.32, and *g*_∥_ = 2.02 and *g*_⊥_ = 2.24, respectively.^43^ Similarly, the triethylamine and isocyanide adducts of **9** exhibit *g*_∥_ = 1.97 and *g*_⊥_ = 2.39, and *g*_∥_ = 2.00 and *g*_⊥_ = 2.16, respectively.^44^

## Conclusions

Herein, general relationships between the magnetic anisotropy (parametrized by the *g*- and ZFS-values) and the degeneracy of the orbital ground levels of three-coordinate transition metal complexes are established. Starting point is a precise determination of the *g*- and ZFS-values of the triplet state of complex **1**. Complementary SQUID magnetometry and FD-FT-THz-EPR spectroscopy reveal *D* = +33.54 cm^–1^, *E* = 3.15 cm^–1^ (*E*/*D* = 0.09), *g*_*z*_ = *g*_∥_ = 1.96 and *g*_*x*_ = *g*_*y*_ = *g*_⊥_ = 2.45. By coupling an EH analysis with wave-function-based CASSCF/NEVPT2 calculations, we show that the pronounced magnetic easy-plane anisotropy of **1** originates from seemingly negligible SOC-induced mixing of the nonrelativistic ground state ^3^A_2g_ with an orbitally nearly doubly degenerate excited state ^3^E_g_ that lies ∼1800 cm^–1^ above ^3^A_2g_. Consequently, the low-lying magnetic triplet contains 2–4% of ^3^E_g_, which results in a large, positive magnetic anisotropy. For the same reason, *g*_⊥_ is substantially greater than 2, and *g*_∥_ is lower than 2. This analysis is extended to related complexes to identify general characteristics of their magneto-structural correlations. Based on this analysis, we propose the criterion in Table 1 to identify the degree of ground-level orbital near-degeneracy by the symmetry of the electronic ***g***-tensor and the sign of *D*.

The significance of the established criteria extends far beyond the characterization of the complexes discussed here. Rather, they can be used to qualitatively characterize the electronic structures of a broad range of transition-metal complexes with low coordination number. Since both the magnetism as well as the chemical properties of metal complexes depend on their electronic structure, the criteria developed herein can make an important contribution to the characterization of novel complexes and may in combination with quantum chemical calculations guide knowledge-based synthesis approaches.

## Supporting Information Available

FD-FT THz-EPR spectra; magnetometry data; details on the derivation of the effective Hamiltonian, and CASSCF(8,12)/NEVPT2 calculations on complexes **1**, **4** and **5** (PDF)
