# Supplementary material for: A Spectroscopic Criterion for Identifying the Degree of Ground-Level Near-Degeneracy Derived from Effective Hamiltonian Analyses of Three-Coordinate Iron Complexes
Source: JACS Au. 2025 Feb 6;5(2):1016–30. doi: 10.1021/jacsau.4c01256 (PMC11862956; doi:10.1021/jacsau.4c01256)
Supplement: Supplementary file 1 — au4c01256_si_001.pdf [file au4c01256_si_001.pdf]

Supporting Information

A Spectroscopic Criterion for Identifying the Degree of Ground-Level Near-Degeneracy Derived from Effective Hamiltonian Analyses of Three-Coordinate Iron Complexes

Wang Chen,[†#‡] Nikolai Kochetov,[§,&‡] Thomas Lohmiller,[&,$] Qing Liu,[‖] Liang Deng,[‖*] Alexander Schnegg,[§&*] Shengfa Ye[†□*]

[†] State Key Laboratory of Catalysis, Dalian Institute of Chemical Physics, Chinese Academy of Sciences, 457 Zhongshan Road, Dalian 116023, China

[§] EPR Research Group, Max Planck Institute for Chemical Energy Conversion, D-45470 Mülheim an der Ruhr, Germany

[&] EPR4Energy Joint Lab, Department Spins in Energy Conversion and Quantum Information Science, Helmholtz-Zentrum Berlin für Materialien und Energie GmbH, 12489 Berlin, Germany

[$] Institut für Chemie, Humboldt–Universität zu Berlin, 12489 Berlin, Germany

[‖] State Key Laboratory of Organometallic Chemistry, Shanghai Institute of Organic Chemistry, University of Chinese Academy of Sciences, Chinese Academy of Sciences, Shanghai 200032, China

[#] University of Chinese Academy of Sciences, Beijing 100049, China

[□]Key Laboratory of Bioinorganic and Synthetic Chemistry of Ministry of Education, Guangdong Basic Research Center of Excellence for Functional Molecular Engineering, School of Chemistry, IGCME, Sun Yat-sen University, Guangzhou 510275, China

[‡] These authors contributed equally.

Table of Contents

FD-FT THz-EPR and magnetometry....3

Effective Hamiltonian Theory 8

CASSCF(8,12)/NEVPT2 calculations on complex **1**, **4** and **5**....17

FD-FT THz-EPR and magnetometry

FD-FT THz-EPR spectra of **1** were recorded at the THz-EPR beam-line at BESSY II using low-α mode coherent synchrotron radiation[1] with the spectrometer aligned in Voigt geometry and with the magnetic-field component B_1 of the THz-radiation oriented perpendicular to the static magnetic field B_0.[2] IR measurements were taken with a Bruker IFS 125 FTIR spectrometer equipped with a 6 μm multilayer beam splitter and a liquid-He-cooled (4.2 K) bolometer detector (Infrared labsTM). The spectrometer was operated with 0.5 cm^{-1} instrumental resolution. THz-EPR spectra in Figure 1 of the main text, Figure S1, S2 and S4 are shown as magnetic field division spectra (MDS), where raw spectra obtained at 4.8 K and an external magnetic field B_0 + 0.5 T are divided by a reference spectrum measured at B_0. In addition, depicted in Figure S1 is a temperature division spectrum (TDS), where a raw spectrum taken at zero field and 4.8 K was divided by a 100 K reference.

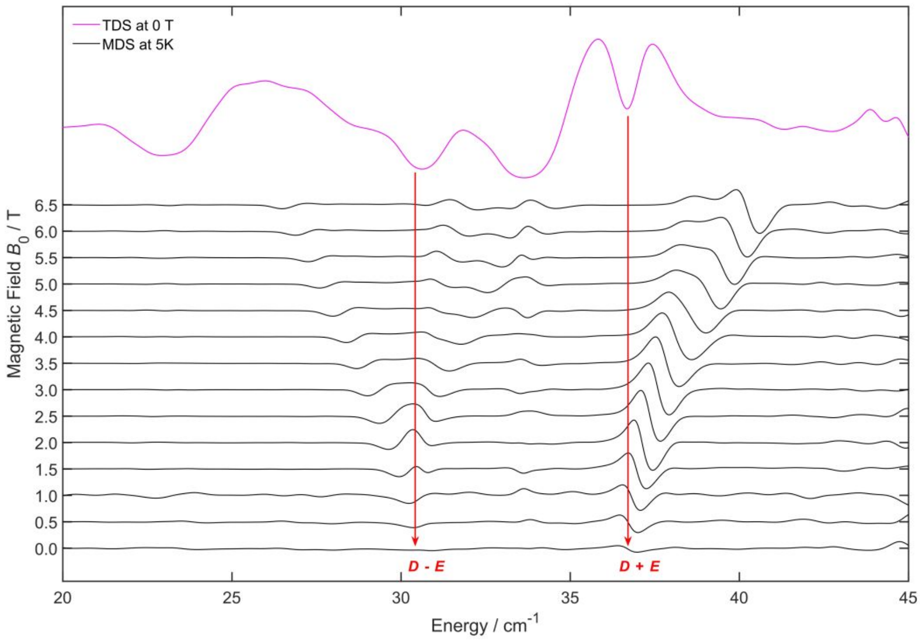

Figure S1. Experimentally obtained THz-EPR spectra of **1**, plotted as TDS 4.8 K/~100 K at 0 T (top, magenta line) and MDS (black lines) at 4.8 K obtained with 0.5 T field steps between 0 T and 7 T (black lines). Red arrows indicate the estimated positions of the ZFS peaks at $|D| - |E|$ and $|D| + |E|$. Distortions in the TDS around 33.5 cm^{-1} are assigned to vibrational modes.

SH parameters of complex **1** were extracted by numerical fits of spectral simulations with the Matlab toolbox EasySpin [3] based on Eq. 1 of the main text to the experimental THz-EPR and SQUID data. Table S1 summarizes the ZFS- and g-values obtained from simulations of the FD-FT-THz-EPR spectra (see Figure 1 c) and d) of the main text), the susceptibility and variable temperature variable field VTVF magnetometry data (Figure 1 a) and b) of the main text), and from the simultaneous simulations of the EPR and magnetometry data (see Figure S2 and S3).

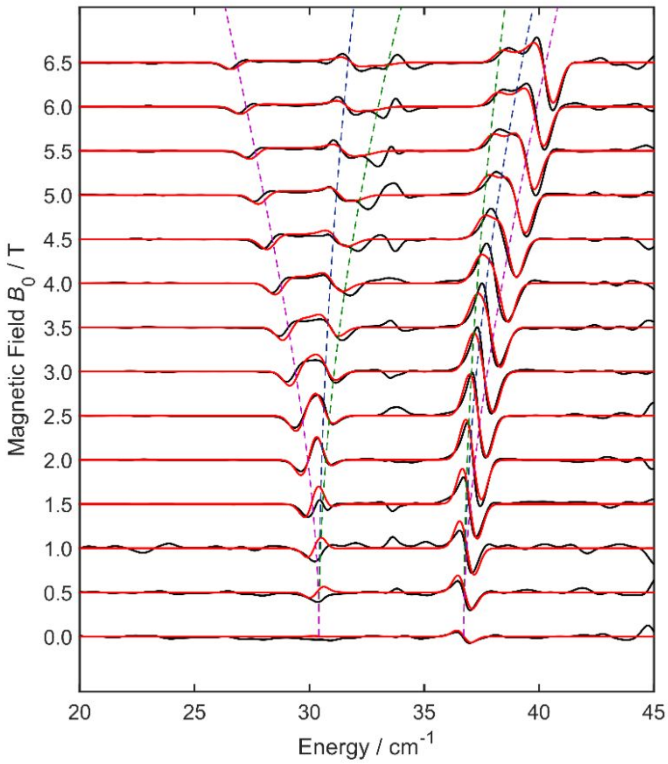

Figure S2. Experimental (black lines) and simulated (red lines) FD-FT THz-EPR MDS at 4.8 K obtained by simultaneous fit of SH parameters with magnetometry data: D = 33.57 cm^{-1}, E/D= 0.09, $g_{\parallel}$ = 1.96, $g_{\perp}$ = 2.28. Dashed lines indicate simulated field-dependent transition energies for $\boldsymbol{B_0}$ aligned along the x- (blue), y- (green) and z-axes (magenta) of the ZFS-tensor.

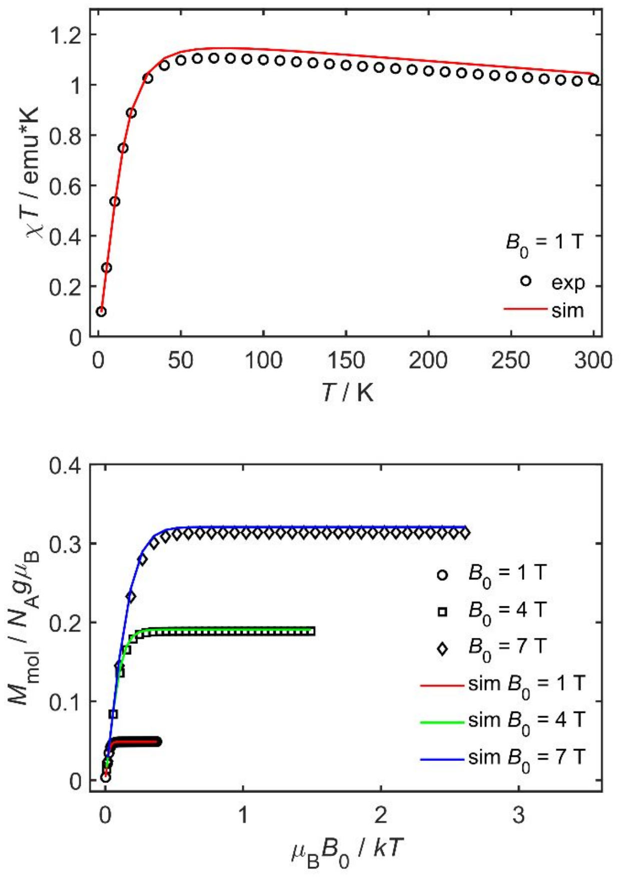

Figure S3. Experimental (dots) and simulated (lines) susceptibility (upper box) and VTVF (lower box) curves obtained by simultaneous fit of SH parameters with FD-FT THz-EPR spectra: D = 33.57 cm^{-1}, E/D= 0.09, $g_{\parallel}$ = 1.96, $g_{\perp}$ = 2.28.

Table S1. Comparison of SH parameters obtained from fits to THz-EPR and SQUID magnetometry data.

Best-fit simulations	D/cm^{-1}	E/cm^{-1}	E/D	g-values
FD-FT THz-EPR	33.54	3.16	0.09	$g_{\perp}$ = 2.45, $g_{\parallel}$ = 1.96
SQUID Magnetometry	33.54	3.16	0.09	$g_{\perp}$ = 2.23, $g_{\parallel}$ = 1.97
Simultaneous fits to THz-EPR spectra and magnetometry data	33.57	3.15	0.09	$g_{\perp}$ = 2.28, $g_{\parallel}$ = 1.96

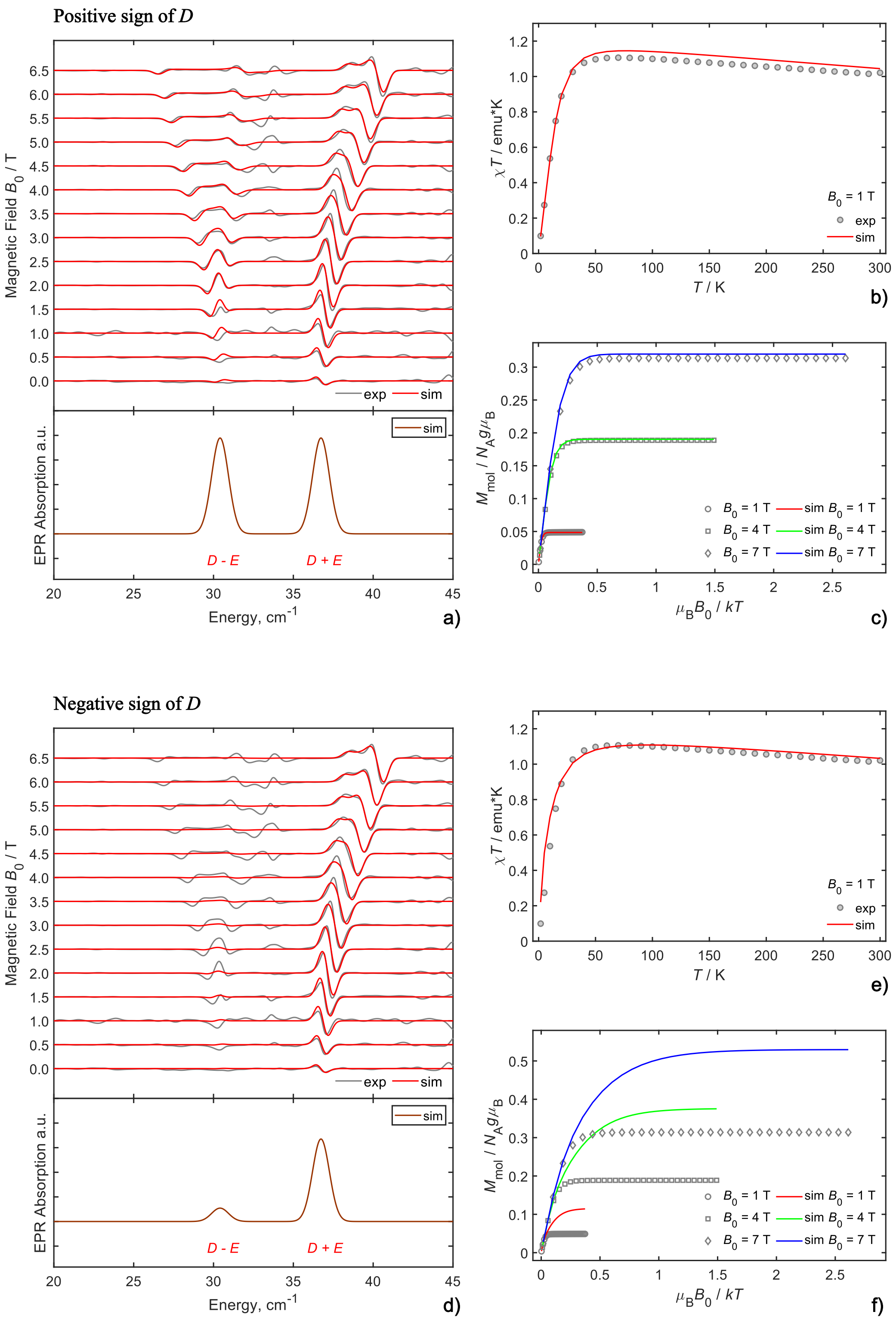

Figure S4 THz-EPR and magnetometry data simulated for a) to c) with D = 33.57 cm^{-1}, E/D= 0.09, $g_{\|}$ = 1.97, $g_{\perp}$ = 2.28 and for d) to e) with the same SH parameters but negative D; a) and d) experimental and simulated THz-EPR MDS at 4.8 K (top panels) and simulated frequency-domain EPR absorption spectra at 0 T and at 4.8 K (bottom panels), b) and e) experimental and simulated magnetic susceptibility curves and c) and f) experimental and simulated VTVF curves.

Figure S4 shows simulations with Eq. 1 assuming positive and negative D-values. The simulations are

overlaid on experimental MDS THz-EPR spectra and magnetometry traces. In Figure S4 a) and d) simulated frequency-domain EPR absorption spectra at zero-field are plotted in the panel below the THz-EPR MDS. Note, the two EPR absorption peaks in Figure S4 correspond to the $D + E$ and $D - E$ peaks marked in the zero-field THz-EPR TDS in Figure S1. For positive D at 4.8 K, the $D + E$ and the $D - E$ transitions have approximalety the same intensity, while for negative *D*, at 4.8 K the $D + E$ transitions are much more intense than the $D - E$ transitions. Clearly, simulations with positive *D* provide better agreement with the experimental data.

The differences in relative intensities in the THz-EPR spectra for negative and positive *D* result from the fact that the energy corresponding to $2E$ is larger than the thermal energy ($2E$ = 6 cm^{-1} corresponds to ~9 K > 4.8 K). Therefore, the relative intensities of the spin transitions from the Boltzmann populated ground-state levels (see e.g. Figure 1 d) in the main text) is sensitive to the sign of *D*. For positive *D* (Figure S4 a)), both $\Delta M_S = \pm 1$ transitions from the $M_S = 0$ ground state have approximately equal intensity. For negative *D* and positive *E*, M_S = -1 is the ground state, which carries most of the spin population at 4.8 K. In this case, the transition from M_S = -1 to M_S = 0 (right branch in Figure S4 d)) would lead to a much more intense EPR absorption as compared to the transitions between the nearly unpopulated M_S = +1 and M_S = 0 levels (left branch Figure S4 d)). Complementary analysis of low temperature THz-EPR and SQUID data, therefore, allows for a clear-cut and independent assignment of the sign and magnitude of the ZFS-values and the anisotropy of the ***g***-tensor.

Effective Hamiltonian Theory

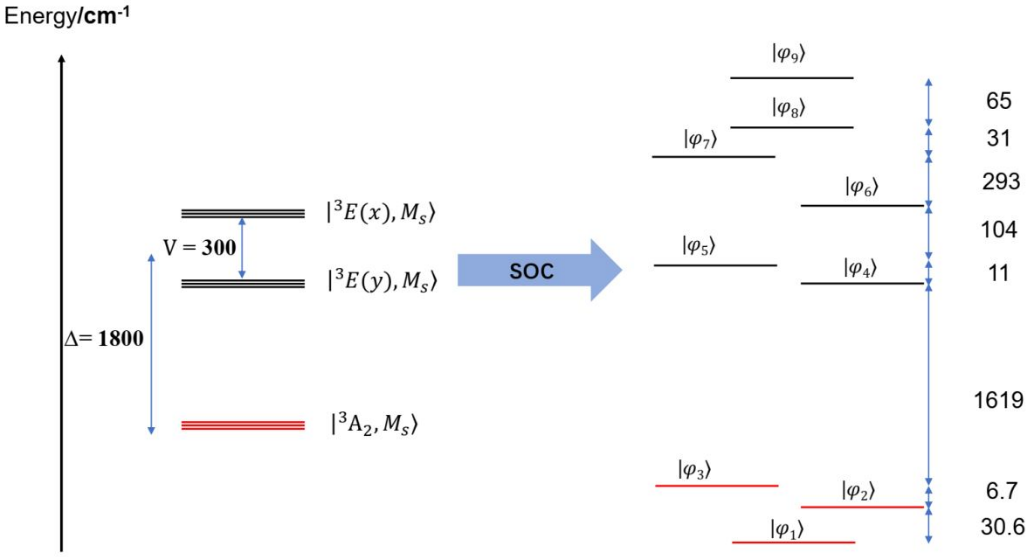

Figure S5. Energy spectrum without (left) and with (right) SOC with Δ = 1800 cm^{-1} and V = 300 cm^{-1}.

To establish magneto-structural correlations, an effective Hamiltonian (EH) approach was applied. The EH (Equations 2a) to 2b) in the main text) is derived in the basis of the low-lying triplet to explicitly consider the SOC and the Zeeman interactions. Diagonalizing the $\hat{\boldsymbol{H}}_{\Delta}$ and $\hat{\boldsymbol{H}}_{\mathbf{SOC}}$ parts of the EH-matrix yields nine eigenfunctions pertaining to nine magnetic sublevels (see Figure S5). Consequently, the SH parameters can be extracted by mapping the lowest three magnetic sublevels obtained from diagonalization of the EH matrix in the basis of the many electron functions (Equations S1a to S1i) to the fictitious pseudo-spin eigenstates M_s = +1, −1 and 0 of $\tilde{\hat{S}}$ = 1.

Many electrons basis function:

$$\left|{}^3\mathrm{A}_{2g}, +1\right\rangle = \left|\mathrm{d}_{z^2}\overline{\mathrm{d}_{z^2}}\mathrm{d}_{yz}\mathrm{d}_{xz}\right| \quad \text{(S1a)}$$

$$\left|{}^3\mathrm{A}_{2g}, -1\right\rangle = \left|\mathrm{d}_{z^2}\overline{\mathrm{d}_{z^2}}\,\overline{\mathrm{d}_{yz}}\,\overline{\mathrm{d}_{xz}}\right| \quad \text{(S1b)}$$

$$\left|{}^3\mathrm{A}_{2g}, 0\right\rangle = \frac{1}{\sqrt{2}}\left(\left|\mathrm{d}_{z^2}\,\overline{\mathrm{d}_{z^2}}\,\overline{\mathrm{d}_{yz}}\,\mathrm{d}_{xz}\right| + \left|\mathrm{d}_{z^2}\,\overline{\mathrm{d}_{z^2}}\mathrm{d}_{yz}\overline{\mathrm{d}_{xz}}\right|\right) \quad \text{(S1c)}$$

$$\left|{}^3\mathrm{E}_{g}(x), +1\right\rangle = \left|\mathrm{d}_{z^2}\mathrm{d}_{yz}\,\overline{\mathrm{d}_{yz}}\,\mathrm{d}_{xz}\right| \quad \text{(S1d)}$$

$$\left|{}^3\mathrm{E}_{g}(x), -1\right\rangle = \left|\overline{\mathrm{d}_{z^2}}\mathrm{d}_{yz}\,\overline{\mathrm{d}_{yz}}\,\overline{\mathrm{d}_{xz}}\right| \quad \text{(S1e)}$$

$$\left|{}^3\mathrm{E}_{g}(x), 0\right\rangle = \frac{1}{\sqrt{2}}\left(\left|\overline{\mathrm{d}_{z^2}}\,\mathrm{d}_{yz}\,\overline{\mathrm{d}_{yz}}\,\mathrm{d}_{xz}\right| + \left|\mathrm{d}_{z^2}\,\mathrm{d}_{yz}\,\overline{\mathrm{d}_{yz}}\,\overline{\mathrm{d}_{xz}}\right|\right) \quad \text{(S1f)}$$

$$\left|{}^3\mathrm{E}_{g}(y), +1\right\rangle = \left|\mathrm{d}_{z^2}\mathrm{d}_{yz}\,\mathrm{d}_{xz}\,\overline{\mathrm{d}_{xz}}\right| \quad \text{(S1g)}$$

$$\left|{}^3\mathrm{E}_{g}(y), -1\right\rangle = \left|\overline{\mathrm{d}_{z^2}}\,\overline{\mathrm{d}_{yz}}\,\mathrm{d}_{xz}\,\overline{\mathrm{d}_{xz}}\right| \quad \text{(S1h)}$$

$$\left|{}^3\mathrm{E}_{g}(y), 0\right\rangle = \frac{1}{\sqrt{2}}\left(\left|\mathrm{d}_{z^2}\,\overline{\mathrm{d}_{yz}}\mathrm{d}_{xz}\,\overline{\mathrm{d}_{xz}}\right| + \left|\overline{\mathrm{d}_{z^2}}\,\mathrm{d}_{yz}\mathrm{d}_{xz}\,\overline{\mathrm{d}_{xz}}\right|\right) \quad \text{(S1i)}$$

The corresponding EH-matrix in the basis of the many electrons basis functions is depicted in Table S3.

Table S2. $\hat{\boldsymbol{H}}_{\mathrm{EH}}$ matrix in the basis of the many electrons basis functions without symmetry constraint. $\hat{\boldsymbol{H}}_{\Delta}$, $\hat{\boldsymbol{H}}_{\mathrm{SOC}}$ and the spin and orbital $\hat{\boldsymbol{H}}_{\mathrm{Zee}}$ terms are displayed in red, green, brown and blue, respectively. B_x, B_y and B_z are the three components of the applied field and $B_{\pm} = \frac{1}{\sqrt{2}}\left(B_x \pm iB_y\right)$. Zero matrix elements are omitted.

$\lvert ^3A_{2g}, 0\rangle$	$\lvert ^3A_{2g}, -1\rangle$	$\lvert ^3A_{2g}, +1\rangle$	$\lvert ^3E_g(x), +1\rangle$	$\lvert ^3E_g(y), +1\rangle$	$\lvert ^3E_g(x), -1\rangle$	$\lvert ^3E_g(y), -1\rangle$	$\lvert ^3E_g(x), 0\rangle$	$\lvert ^3E_g(y), 0\rangle$
0	$2\mu_B B_-$	$2\mu_B B_+$	$-\frac{\sqrt{6}}{4}\zeta i$	$\frac{\sqrt{6}}{4}\zeta$	$-\frac{\sqrt{6}}{4}\zeta i$	$-\frac{\sqrt{6}}{4}\zeta$	$\sqrt{3}\mu_B B_x i$	$\sqrt{3}\mu_B B_y i$
$2\mu_B B_+$	$-2\mu_B B_z$				$\sqrt{3}\mu_B B_x i$	$\sqrt{3}\mu_B B_y i$	$-\frac{\sqrt{6}}{4}\zeta i$	$\frac{\sqrt{6}}{4}\zeta$
$2\mu_B B_-$		$2\mu_B B_z$	$\sqrt{3}\mu_B B_x i$	$\sqrt{3}\mu_B B_y i$			$-\frac{\sqrt{6}}{4}\zeta i$	$-\frac{\sqrt{6}}{4}\zeta$
$\frac{\sqrt{6}}{4}\zeta i$		$-\sqrt{3}\mu_B B_x i$	$\Delta+V/2+2\mu_B B_z$	$\frac{1}{2}\zeta i-\mu_B B_z i$			$2\mu_B B_-$	
$\frac{\sqrt{6}}{4}\zeta$		$-\sqrt{3}\mu_B B_y i$	$-\frac{1}{2}\zeta i+\mu_B B_z i$	$\Delta-V/2+2\mu_B B_z$				$2\mu_B B_-$
$\frac{\sqrt{6}}{4}\zeta i$	$-\sqrt{3}\mu_B B_x i$				$\Delta+V/2-2\mu_B B_z$	$-\frac{1}{2}\zeta i-\mu_B B_z i$	$2\mu_B B_+$	
$-\frac{\sqrt{6}}{4}\zeta$	$-\sqrt{3}\mu_B B_y i$				$\frac{1}{2}\zeta i+\mu_B B_z i$	$\Delta-V/2-2\mu_B B_z$		$2\mu_B B_+$
$-\sqrt{3}\mu_B B_x i$	$\frac{\sqrt{6}}{4}\zeta i$	$\frac{\sqrt{6}}{4}\zeta i$	$2\mu_B B_+$		$2\mu_B B_-$		$\Delta+V/2$	$-\mu_B B_z i$
$-\sqrt{3}\mu_B B_y i$	$\frac{\sqrt{6}}{4}\zeta$	$-\frac{\sqrt{6}}{4}\zeta$		$2\mu_B B_+$		$2\mu_B B_-$	$\mu_B B_z i$	$\Delta-V/2$

In the following the procedure to derive SH parameters without any symmetry constraints is described (for more details of this analysis see Chibotaru and Ungur[4]).

As shown in Table S3, the lowest three magnetic sublevels (φ_1, φ_2 and φ_3) are obtained by diagonalization of the $\hat{H}_{\Delta}$ and $\hat{H}_{\mathrm{SOC}}$ parts of the EH-matrix. When Δ = 1800 cm^{-1} and V = 300 cm^{-1}, their components are given by

$$|\varphi_1\rangle = 0.9816\left|^3A_{2g},0\right\rangle -0.1034i\left|^3E_g(x),+1\right\rangle -0.0868\left|^3E_g(y),+1\right\rangle -0.1034i\left|^3E_g(x),-1\right\rangle +0.0868\left|^3E_g(y),+1\right\rangle \quad \text{(S2a)}$$

$$|\varphi_2\rangle = (-0.6944-0.0696i)\left|^3A_{2g},-1\right\rangle + (-0.6944-0.0696i)\left|^3A_{2g},+1\right\rangle + (-0.0161+0.1605i)\left|^3E_g(x),0\right\rangle \quad \text{(S2b)}$$

$$|\varphi_3\rangle = 0.7003\left|^3\mathrm{A}_{2g}, -1\right\rangle - 0.7003\left|^3\mathrm{A}_{2g}, +1\right\rangle - 0.1380\left|^3\mathrm{E}_g(y), 0\right\rangle \quad \text{(S2c)}$$

Now the constraints imposed by the time inversion relations need to be considered. For a true $\hat{S}=1$ system, the following relations are valid: $\hat{\theta}|1, +1\rangle = |1, -1\rangle$, $\hat{\theta}|1, -1\rangle = |1, +1\rangle$ and $\hat{\theta}|1, 0\rangle = -|1, 0\rangle$ ($\hat{\theta}$ is the time inverse operator). Wave functions φ_a, φ_b and φ_c fulfilling the time inversion relations can be obtained by linear combination of φ_1, φ_2 and φ_3. Then the magnetic moment matrices $\boldsymbol{\mu_\alpha}$ ($\alpha = x,y,z$) along B_x, B_y and B_z in the basis of φ_a, φ_b and φ_c can be obtained.

$$\boldsymbol{\mu_x} = \begin{pmatrix} 1.5699 & -0.6292-1.2326i & 0 \\ -0.6292+1.2326i & 0 & -0.6292-1.2326i \\ 0 & -0.6292+1.2326i & -1.5699 \end{pmatrix}\mu_B \quad \text{(S3a)}$$

$$\boldsymbol{\mu_y} = \begin{pmatrix} 1.4648 & -0.5871+1.2326i & 0 \\ -0.5871-1.2326i & 0 & -0.5871+1.2326i \\ 0 & -0.5871-1.2326i & -1.4648 \end{pmatrix}\mu_B \quad \text{(S3b)}$$

$$\boldsymbol{\mu_z} = \begin{pmatrix} 0.9750 & 1.2163 & 0 \\ 1.2163 & 0 & 1.2163 \\ 0 & 1.2163 & -0.9750 \end{pmatrix}\mu_B \quad \text{(S3c)}$$

For pseudospin $\tilde{\hat{S}} = 1$, the magnetic moment matrix $\boldsymbol{\mu_\alpha}$ is generally parametrized as follows:

$$\boldsymbol{\mu_x} = \begin{pmatrix} g_{xz} & (g_{xx}-ig_{xy})/\sqrt{2} & 0 \\ (g_{xx}+ig_{xy})/\sqrt{2} & 0 & (g_{xx}-ig_{xy})/\sqrt{2} \\ 0 & (g_{xx}+ig_{xy})/\sqrt{2} & -g_{xz} \end{pmatrix}\mu_\mathrm{B} \quad \text{(S4a)}$$

$$\boldsymbol{\mu_y} = \begin{pmatrix} g_{yz} & (g_{yx}-ig_{yy})/\sqrt{2} & 0 \\ (g_{yx}+ig_{yy})/\sqrt{2} & 0 & (g_{yx}-ig_{yy})/\sqrt{2} \\ 0 & (g_{yx}+ig_{yy})/\sqrt{2} & -g_{yz} \end{pmatrix}\mu_\mathrm{B} \quad \text{(S4b)}$$

$$\boldsymbol{\mu_z} = \begin{pmatrix} g_{zz} & (g_{zx}-ig_{zy})/\sqrt{2} & 0 \\ (g_{zx}+ig_{zy})/\sqrt{2} & 0 & (g_{zx}-ig_{zy})/\sqrt{2} \\ 0 & (g_{zx}+ig_{zy})/\sqrt{2} & -g_{zz} \end{pmatrix}\mu_\mathrm{B} \quad \text{(S4c)}$$

Therefore, the corresponding $\boldsymbol{g}$ and $\boldsymbol{G} = \boldsymbol{g}^*\boldsymbol{g}^\mathrm{T}$ ($\boldsymbol{G}$, Abragam-Bleaney tensor) matrices can be achieved:

$$\boldsymbol{g} = \begin{pmatrix} -0.8898 & -1.7431 & 1.5699 \\ -0.8303 & 1.7431 & 1.4448 \\ 1.7201 & 0 & 0.9750 \end{pmatrix} \quad \text{(S5a)}$$

$$\boldsymbol{G} = \begin{pmatrix} G_x & 0 & 0 \\ 0 & G_y & 0 \\ 0 & 0 & G_y \end{pmatrix} = \begin{pmatrix} 6.2948 & 0 & 0 \\ 0 & 5.8734 & 0 \\ 0 & 0 & 3.9092 \end{pmatrix} \quad \text{(S5b)}$$

$$\boldsymbol{g} = \begin{pmatrix} \sqrt{G_x} & 0 & 0 \\ 0 & \sqrt{G_y} & 0 \\ 0 & 0 & \sqrt{G_y} \end{pmatrix} = \begin{pmatrix} 2.5089 & 0 & 0 \\ 0 & 2.4235 & 0 \\ 0 & 0 & 1.9772 \end{pmatrix} \quad \text{(S5c)}$$

that yield the principle g-values: $g_x = 2.5089$, $g_y = 2.4235$ and $g_z = 1.9772$. In addition, the off-diagonal elements of the $\boldsymbol{G}$-matrix being zero suggest that the principal coordinate system of the $\boldsymbol{g}$-tensor coincides with the molecular coordinate system. This is consistent with the made assumptions, since the coordinate axes are defined based on the non-bonding orbitals d_{z^2}, d_{xz} and d_{yz} and do not change in the following process. In other words, we identify the main magnetic axes and take it as the molecular coordinate system based on the orbital angular momentum operators ($\hat{L}_{x,y,z}$). φ_0, φ_+ and φ_- corresponding to $|1,0\rangle$, $|1,+1\rangle$ and $|1,-1\rangle$ are derived by diagonalization of the magnetic-moments matrix along the main magnetic z-axis.

$$|\varphi_0\rangle = -0.9816\left|{}^3\mathrm{A}_{2g},0\right\rangle + 0.1034i\left|{}^3\mathrm{E_g}(x),+1\right\rangle + 0.0817\left|{}^3\mathrm{E_g}(y),+1\right\rangle$$

$$+0.0961i\left|{}^3\mathrm{E_g}(x),-1\right\rangle - 0.0817\left|{}^3\mathrm{E_g}(y),+1\right\rangle \quad \text{(S6a)}$$

$$|\varphi_+\rangle = (-0.7111 + 0.6896i)\left|{}^3\mathrm{A}_{2g},+1\right\rangle + (0.0793 + 0.0820i)\left|{}^3\mathrm{E_g}(x),0\right\rangle$$

$$+ (0.0013 - 0.0012i)\left|{}^3\mathrm{A}_{2g},-1\right\rangle + (-0.0702 + 0.0678i)|{}^3\mathrm{E}(y),0\rangle \quad \text{(S6b)}$$

$$|\varphi_-\rangle = (0.0013 + 0.0012i)\left|{}^3\mathrm{A}_{2g},+1\right\rangle + (-0.0793 + 0.0820i)\left|{}^3\mathrm{E_g}(x),0\right\rangle$$

$$+ (-0.7111 - 0.6896i)\left|{}^3\mathrm{A}_{2g},-1\right\rangle + (0.0702 + 0.0678i)\left|{}^3\mathrm{E_g}(y),0\right\rangle \quad \text{(S6c)}$$

The relative energies of the lowest three magnetic sublevels φ_i (i = 1, 2, 3) are $E_i' = E_i - E_0$, $E_0 = (E_1 + E_2 + E_3)/3$. The corresponding ZFS-Hamiltonian is written as follows:

$$\hat{H}_{\mathrm{ZFS}} = E_1'|\varphi_1\rangle\langle\varphi_1| + E_2'|\varphi_2\rangle\langle\varphi_2| + E_3'|\varphi_3\rangle\langle\varphi_3|$$
(S7a)

Because the wave functions φ_1, φ_2 and φ_3 can be composed of φ_0, φ_+ and φ_- as follows:

$$|\varphi_1\rangle = |\varphi_0\rangle \quad \text{(S8a)}$$

$$|\varphi_2\rangle = (0.4554 + 0.5376i)|\varphi_+\rangle + (0.5531 - 0.4365i)|\varphi_-\rangle \quad \text{(S8b)}$$

$$|\varphi_3\rangle = (0.5104 + 0.4930i)|\varphi_+\rangle + (-0.5104 + 0.4930i)|\varphi_-\rangle \quad \text{(S8c)}$$

Therefore, we can write the ZFS-Hamiltonian as a 3 × 3 matrix in the basis of φ_0, φ_+ and φ_-. Meanwhile, the ZFS-Hamiltonian matrix can be decomposed in $|1,0\rangle$, $|1,+1\rangle$ and $|1,-1\rangle$ for pseudospin $\tilde{\hat{S}} = 1$:

$$\hat{H}_{\mathrm{ZFS}} = \tilde{\hat{S}}\boldsymbol{D}\tilde{\hat{S}} \quad \text{(S9a)}$$

As a consequence, the following *D*-matrix is obtained:

$$\boldsymbol{D} = \begin{pmatrix} -11.4428 & -3.4198 & 0 \\ -3.4198 & -11.2058 & 0 \\ 0 & 0 & 22.6486 \end{pmatrix}$$

(S10a)

$$\boldsymbol{U} = \begin{pmatrix} 0.6948 & -0.7192 & 0 \\ -0.7192 & -0.6948 & 0 \\ 0 & 0 & 1 \end{pmatrix} \quad \text{(S10b)}$$

$$\boldsymbol{U}^{-\mathbf{1}}\boldsymbol{D}\boldsymbol{U} = \begin{pmatrix} D_{xx} & 0 & 0 \\ 0 & D_{yy} & 0 \\ 0 & 0 & D_{zz} \end{pmatrix} = \begin{pmatrix} -7.9024 & 0 & 0 \\ 0 & -14.7461 & 0 \\ 0 & 0 & 22.6486 \end{pmatrix} \quad \text{(S10c)}$$

Hence, $D = \frac{3}{2}D_{zz} = 33.97\ \mathrm{cm}^{-1}$, $E = \frac{1}{2}(D_{xx} - D_{yy}) = 3.42\ \mathrm{cm}^{-1}$ are obtained. On top of this, the main anisotropy axes is obtained by rotating the molecular coordinate system with the unitary transformation *U*. In the current situation, the *U*-matrix means rotation clockwise 46.0 degrees around the *Z*-axis. Figure S6 depicts the ***g***-tensor axes and the ***D***-tensor anisotropy axes derived from the EH approach.

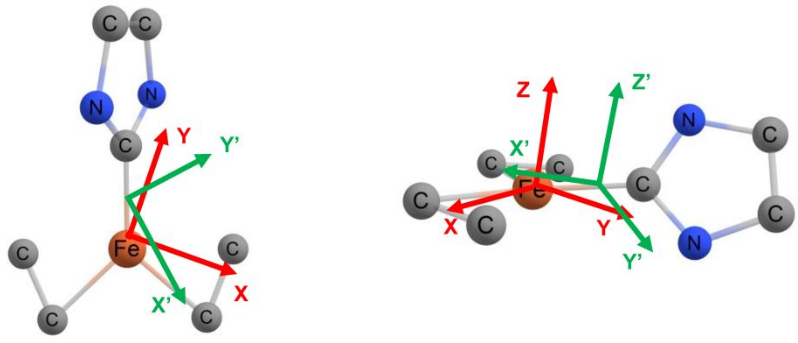

Figure S6. Two views on the molecular structure of **1** of which all substituents on carbene and alkene are omitted for clarity. The ***g***-tensor axes (*x, y, z*) (Red arrows) and ***D***-tensor axes (*x', y', z'*) (Green arrows) obtained from the EH analysis.

The $^3A_{2g}$ ground state is orbitally nondegenerate, whereas the ^3E_g excited states feature unquenched orbital angular momentum along the z-direction. As elaborated in our earlier work,[5] the resulting first-order SOC, so called in-state SOC, leads to three accidentally degenerate doublets, which are the eigenstates of the L_z and S_z operators given by

$$\left|{}^3\mathrm{E_g}, M_S, +1\right\rangle = \frac{1}{\sqrt{2}}\left(|\mathrm{E_g}(x), M_S\rangle - i|\mathrm{E_g}(y), M_S\rangle\right) \quad \text{(S11a)}$$

$$\left|{}^3\mathrm{E_g}, M_S, -1\right\rangle = \frac{1}{\sqrt{2}}\left(|\mathrm{E_g}(x), M_S\rangle + i|\mathrm{E_g}(y), M_S\rangle\right) \quad \text{(S11b)}$$

$$\left|{}^3\mathrm{A_{2g}}, 0, 0\right\rangle = \left|{}^3\mathrm{A_{2g}}, 0\right\rangle \quad \text{(S11c)}$$

$$\left|{}^3\mathrm{A_{2g}}, \pm 1, 0\right\rangle = \left|{}^3\mathrm{A_{2g}}, \pm 1\right\rangle$$

(S11d)

$$\left\langle {}^3\mathrm{E_g}, M_S, \pm 1 \middle| L_z \middle| {}^3\mathrm{E_g}, \mathrm{M}_S, \pm 1\right\rangle = \pm 1$$

(S11e)

$$\left\langle {}^3\mathrm{E_g}, M_S, \pm 1 \middle| S_z \middle| {}^3\mathrm{E_g}, M_S, \pm 1\right\rangle = M_s$$

(S11f)

$$\left\langle {}^3\mathrm{E_g}, M_S, \pm 1 \middle| H_{\mathrm{SOC}} \middle| {}^3\mathrm{E_g}, M_S, \pm 1\right\rangle = \pm \zeta M_s/2$$

(S11g)

According to the Clebsch–Gordan coefficients of the D_3 double group, the symmetry-adapted states resulting from in-state SOC of ^3E_g and $^3A_{2g}$ can be expressed as:

$$\left|{}^3\mathrm{E_g}, \mathrm{A_1}\right\rangle = \frac{1}{\sqrt{2}}\left(\left|{}^3\mathrm{E_g}, +1, -1\right\rangle + \left|{}^3\mathrm{E_g}, -1, +1\right\rangle\right)$$

(S12a)

$$\left|{}^3\mathrm{E_g}, \mathrm{A_2}\right\rangle = \frac{1}{\sqrt{2}}\left(\left|{}^3\mathrm{E_g}, +1, -1\right\rangle - \left|{}^3\mathrm{E_g}, -1, +1\right\rangle\right)$$

(S12b)

$$\left|{}^3\mathrm{E_g}, \mathrm{E_\pm}\right\rangle = \pm\left|{}^3\mathrm{E_g}, 0, \pm 1\right\rangle \quad \text{(S12c)}$$

$$\left|{}^3\mathrm{E_g}, \mathrm{E_1}\right\rangle = \left|{}^3\mathrm{E_g}, -1, -1\right\rangle$$

(S12d)

$$\left|{}^3\mathrm{E_g}, \mathrm{E_2}\right\rangle = -\left|{}^3\mathrm{E_g}, +1, +1\right\rangle$$

(S12e)

$\left|{}^3A_{2g},A_1\right\rangle = \left|{}^3A_{2g},0,0\right\rangle$ (S12f)

$\left|{}^3A_{2g},E_\pm\right\rangle = \left|{}^3A_{2g},\pm 1,0\right\rangle$

(S12g)

Comparsion to *D* values computed by second-order perturbation treatment and EH theory

The *D*-value can be estimated by using the second order perturbation theory. The energy decrements of $|{}^3A_{2g},E_\pm\rangle$ and $|{}^3A_{2g},A_1\rangle$ resulting from the out-of-state SOC between ${}^3A_{2g}$ and ${}^3E_g$ can be estimated as follows,

$$E^2\left(|{}^3A_{2g},E_\pm\rangle\right) = \frac{\left|\langle {}^3A_{2g},E_\pm \,|\, \hat{\boldsymbol{H}}_{SOC} \,|\, {}^3E,E_\pm\rangle\right|^2}{\Delta E} \quad \text{(S13a)}$$

$$E^2\left(|{}^3A_{2g},A_1\rangle\right) = \frac{\left|\langle {}^3A_{2g},A_1 \,|\, \hat{\boldsymbol{H}}_{SOC} \,|\, {}^3E,A_1\rangle\right|^2}{\Delta E} \quad \text{(S13b)}$$

$$D = E^2\left(|{}^3A_{2g},A_1\rangle\right) - E^2\left(|{}^3A_{2g},E_\pm\rangle\right) \quad \text{(S13c)}$$

For Δ = 1800 cm^{-1}, $D = E^2\left(|{}^3A_{2g},A_1\rangle\right) - E^2\left(|{}^3A_{2g},E_\pm\rangle\right) = 42.7\ \mathrm{cm}^{-1}$. This large positive value reflects that the magnetic anisotropy of **4** originates from strong SOC between ${}^3A_2$ and ${}^3E$.

As illustrated in Figure 4, as Δ/ζ decreases down to 1.2, *D* rises owing to the increasingly enhanced SOC between ${}^3A_{2g}$ and ${}^3E_g$. Intriguingly, EH analyses reveal that further lowering Δ/ζ leads to *D* steadily descending. This behavior cannot be explained by using perturbation theory, which predicts that *D* escalates to infinity as Δ approaches zero. In fact, the *D*-value measures the relative strength of the out-of-state SOC between $|{}^3E_g,A_1\rangle$ and $|{}^3A_{2g},A_1\rangle$ compared to that between $|{}^3E_g,E_\pm\rangle$ and $|{}^3A_{2g},E_\pm\rangle$. As Δ/ζ decreases from 1.2, the latter interaction increases more rapidly than the former; therefore, *D* peaks at Δ/ζ = 1.2.

Table S3. $\hat{\boldsymbol{H}}_{\mathrm{EH}}$ matrix in the basis of symmetry-adapted $^3\mathrm{E_g}$ states and $^3\mathrm{A_{2g}}$ states of the D_3 point double group. The $\hat{\boldsymbol{H}}_{\Delta}$, $\hat{\boldsymbol{H}}_{\mathrm{SOC}}$, spin and orbital $\hat{\boldsymbol{H}}_{\mathrm{Zee}}$ terms are displayed in red, green, brown and blue, respectively. $B_\mathrm{x}, B_\mathrm{y}$ and B_z are the three component of the applied field and $B_{\pm} = \frac{1}{\sqrt{2}}\left(B_\mathrm{x} \pm iB_y\right)$. Zero matrix elements are omitted.

$\vert ^3\mathrm{A}_2, \mathrm{A}_1\rangle$	$\vert ^3\mathrm{A}_2, \mathrm{E}_-\rangle$	$\vert ^3\mathrm{A}_2, \mathrm{E}_+\rangle$	$\vert ^3\mathrm{E_g}, \mathrm{E}_1\rangle$	$\vert ^3\mathrm{E_g}, \mathrm{E}_2\rangle$	$\vert ^3\mathrm{E_g}, \mathrm{E}_-\rangle$	$\vert ^3\mathrm{E_g}, \mathrm{E}_+\rangle$	$\vert ^3\mathrm{E_g}, \mathrm{A}_1\rangle$	$\vert ^3\mathrm{E_g}, \mathrm{A}_2\rangle$
	$2\mu_\mathrm{B}\mathrm{B}_-$	$2\mu_\mathrm{B}\mathrm{B}_+$			$-\sqrt{3}i\mu_\mathrm{B}\mathrm{B}_-$	$\sqrt{3}i\mu_\mathrm{B}\mathrm{B}_+$	$-\frac{\sqrt{6}}{2}\zeta i$	
$2\mu_\mathrm{B}\mathrm{B}_+$	$-2\mu_\mathrm{B}\mathrm{B}_z$		$\sqrt{3}i\mu_\mathrm{B}\mathrm{B}_-$		$\frac{\sqrt{3}}{2}\zeta i$		$\frac{\sqrt{6}}{2}i\mu_\mathrm{B}\mathrm{B}_+$	$-\frac{\sqrt{6}}{2}i\mu_\mathrm{B}\mathrm{B}_+$
$2\mu_\mathrm{B}\mathrm{B}_-$		$2\mu_\mathrm{B}\mathrm{B}_z$		$-\sqrt{3}i\mu_\mathrm{B}\mathrm{B}_+$		$-\frac{\sqrt{3}}{2}\zeta i$	$\frac{\sqrt{6}}{2}i\mu_\mathrm{B}\mathrm{B}_-$	$\frac{\sqrt{6}}{2}i\mu_\mathrm{B}\mathrm{B}_-$
	$-\sqrt{3}i\mu_\mathrm{B}\mathrm{B}_+$		$\Delta-\frac{1}{2}\zeta-2\mu_\mathrm{B}\mathrm{B}_z-\mu_\mathrm{B}\mathrm{B}_z$		$-2\mu_\mathrm{B}\mathrm{B}_+$			
		$\sqrt{3}i\mu_\mathrm{B}\mathrm{B}_-$		$\Delta-\frac{1}{2}\zeta+2\mu_\mathrm{B}\mathrm{B}_z+\mu_\mathrm{B}\mathrm{B}_z$		$-2\mu_\mathrm{B}\mathrm{B}_-$		
$\sqrt{3}i\mu_\mathrm{B}\mathrm{B}_+$	$-\frac{\sqrt{3}}{2}\zeta i$		$-2\mu_\mathrm{B}\mathrm{B}_-$		$\Delta-\mu_\mathrm{B}\mathrm{B}_z$		$-\sqrt{2}\mu_\mathrm{B}\mathrm{B}_+$	$-\sqrt{2}\mu_\mathrm{B}\mathrm{B}_+$
$-\sqrt{3}i\mu_\mathrm{B}\mathrm{B}_-$		$\frac{\sqrt{3}}{2}\zeta i$		$-2\mu_\mathrm{B}\mathrm{B}_+$		$\Delta+\mu_\mathrm{B}\mathrm{B}_z$	$\sqrt{2}\mu_\mathrm{B}\mathrm{B}_-$	$-\sqrt{2}\mu_\mathrm{B}\mathrm{B}_-$
$\frac{\sqrt{6}}{2}\zeta i$	$-\frac{\sqrt{6}}{2}i\mu_\mathrm{B}\mathrm{B}_-$	$-\frac{\sqrt{6}}{2}i\mu_\mathrm{B}\mathrm{B}_+$			$-\sqrt{2}\mu_\mathrm{B}\mathrm{B}_-$	$\sqrt{2}\mu_\mathrm{B}\mathrm{B}_+$	$\Delta+\frac{1}{2}\zeta$	$2\mu_\mathrm{B}\mathrm{B}_z$ $-\mu_\mathrm{B}\mathrm{B}_z$
	$\frac{\sqrt{6}}{2}i\mu_\mathrm{B}\mathrm{B}_-$	$-\frac{\sqrt{6}}{2}i\mu_\mathrm{B}\mathrm{B}_+$			$-\sqrt{2}\mu_\mathrm{B}\mathrm{B}_-$	$-\sqrt{2}\mu_\mathrm{B}\mathrm{B}_+$	$2\mu_\mathrm{B}\mathrm{B}_z$ $-\mu_\mathrm{B}\mathrm{B}_z$	$\Delta+\frac{1}{2}\zeta$

CASSCF(8,12)/NEVPT2 calculations on complexes 1, 4 and 5.

Table S4. Transition energies and SH parameters of the complex **1** estimated by CASSCF(8,12)/NEVPT2 with varying numbers of excited states.

Spin multiplicity	Roots	ΔE/ cm^{-1} of Excited state (S =1)				D/cm^{-1}	E/D	*g-values*
		State 1	State 2	State 3	State 4			
3	3	2270	2400	—	—	34.7	0.0562	2.45, 2.40, 1.98
3	4	2240	2370	16110	—	35.0	0.0680	2.46, 2.40, 1.98
1, 3	6, 10	2410	2560	16250	18250	29.1	0.0765	2.44, 2.39, 1.99
1, 3	11,12	2420	2590	16140	18100	30.0	0.0703	2.44, 2.39, 1.99
1, 3	20,45	2290	2420	16400	18500	29.4	0.0679	2.45, 2.41, 1.99

To validate our perspective of electronic structures of **4, 4'** and **5**, we carried out CASSCF(6,10)/NEVPT2 calculations. As shown in Figure S8-S9, the active space is composed of 6 electrons distributed into orbitals including the five 3d and five 4d orbitals of the Fe center. In analogy to the ligand field analysis in the manuscript, for **4**, and **5,** the forth and fifth excited states both feature doubly occupied d_{xy} and $d_{x^2-y^2}$, and are much higher than the second and third excited states in the energy due to the σ donation of supporting ligand. Furthermore, the second and third excited states feature an electron configuration with doubly occupied d_{xz} and d_{yz}. More importantly, the energies of the second and third exited states both fall into the uncertainty range of wavefunction based *ab initio* quantum chemical calculations and hence are not very reliable. As a consequence, these results indicate these complexes possess near-degenerate ground levels. Although the energies of the second and third excited states are not reliable, these calculations qualitatively support our analysis in the manuscript. For complex **4**, the second and third excited states are close in energy (V/Δ = 0.65) leading to a positive D value and $g_{\perp} > 2 > g_{\parallel}$, and therefore possesse a type I triply degenerate ground level in analogy to **1**. While for complex **5**, because of the π back-bonding interaction between the π* orbital of the NHC ligand and d_{yz} orbital of Fe center, the second excited states is much lower than the third in energy (V/Δ = 1.3) along with a negative D value and $g_x >> g_y > 2 > g_z$. Hence, complex **5** is best interpreted as possessing a type II triply degenerate ground level.

Table S5. SH parameters predicted by CASSCF(6,10)/NEVPT2 for **4** and **5** with varying numbers of excited states.

	Roots (S = 2)	D/cm^{-1}	E/D	g-values
Complex **4**	3	20.6	0.20	2.57, 2.41, 1.95
	5	21.7	0.28	2.64, 2.43, 1.93
Complex **5**	3	-27.3	0.11	2.63, 2.14, 1.97
	5	-31.0	0.10	2.70, 2.14, 1.95

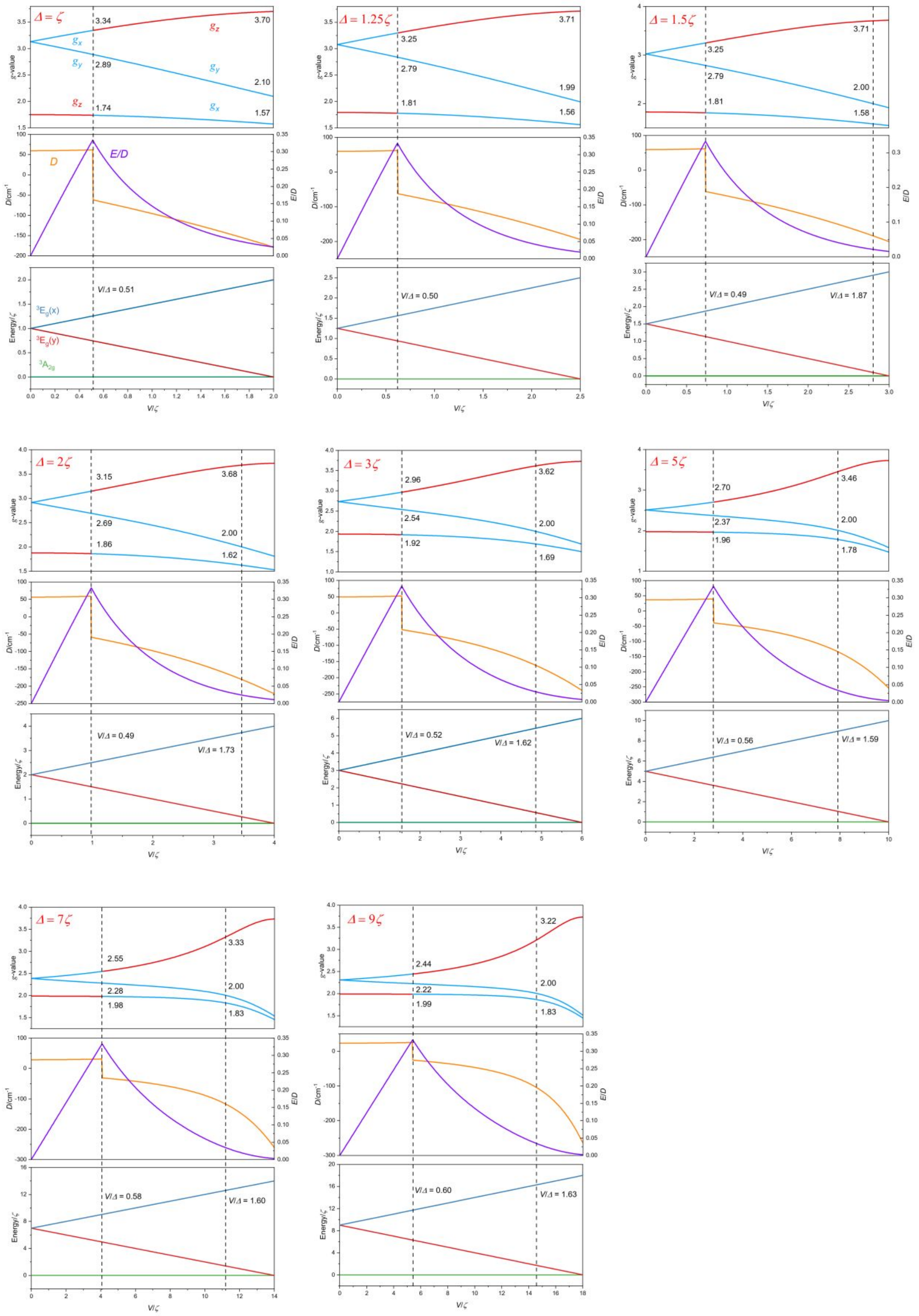

Figure S7. From top to bottom: g-values (red for g_z and blue for $g_{x,y}$), E/D (purple), D (orange), relative energies of low-lying electronic triplet $^3A_{2g}$ (green), $^3E_g(x)$ (blue) and $^3E_g(y)$ (red) as a function of V/ζ with $\zeta = 320$ cm^{-1}. The left and right black dashed lines indicate the situations in which the three lowest states $|\varphi_i\rangle$ have equal distances on the energy axis and g_y equals 2.0, respectively.

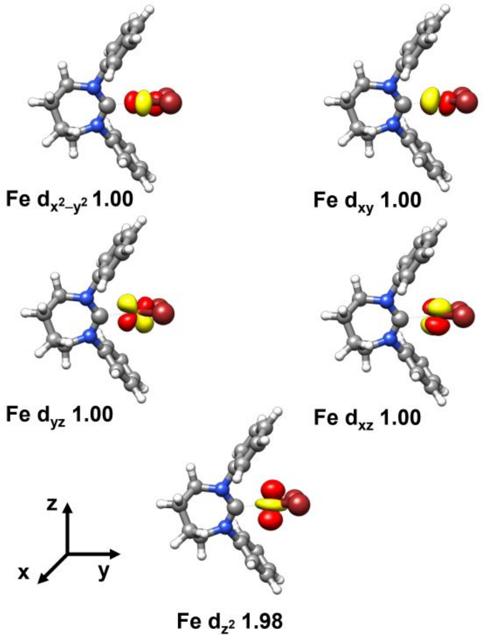

Predominant electron configurations for sates (S=2)	NEVPT2 3 roots /cm^{-1}	NEVPT2 5 roots /cm^{-1}
$(d_{z^2})^2(d_{yz})^1(d_{xz})^1(d_{x^2-y^2})^1(d_{xy})^1$	0	0
$(d_{z^2})^1(d_{yz})^2(d_{xz})^1(d_{x^2-y^2})^1(d_{xy})^1$	530	420
$(d_{z^2})^1(d_{yz})^1(d_{xz})^2(d_{x^2-y^2})^1(d_{xy})^1$	1060	970
$(d_{z^2})^1(d_{yz})^1(d_{xz})^1(d_{x^2-y^2})^1(d_{xy})^2$	—	4240
$(d_{z^2})^1(d_{yz})^1(d_{xz})^1(d_{x^2-y^2})^2(d_{xy})^1$	—	6800

Figure S8. Active orbitals of the ground state of **4** obtained by CASSCF(6,10) calculations. The five second d shell orbitals are omitted. The occupation number of each orbital e are listed near the label. The energies of $S = 2$ states predicted by CASSCF(6,10)/NEVPT2 calculations are listed in the table.

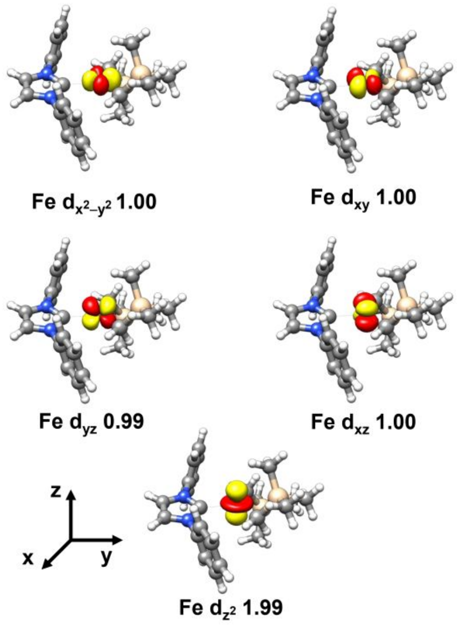

Sates (S=2)	CASSCF energies /cm^{-1}	NEVPT2 energies /cm^{-1}
$(d_{z^2})^2(d_{yz})^1(d_{xz})^1(d_{x^2-y^2})^1(d_{xy})^1$	0	0
$(d_{z^2})^1(d_{yz})^2(d_{xz})^1(d_{x^2-y^2})^1(d_{xy})^1$	490	550
$(d_{z^2})^1(d_{yz})^1(d_{xz})^2(d_{x^2-y^2})^1(d_{xy})^1$	2330	2470
$(d_{z^2})^1(d_{yz})^1(d_{xz})^1(d_{x^2-y^2})^1(d_{xy})^2$	6480	6910
$(d_{z^2})^1(d_{yz})^1(d_{xz})^1(d_{x^2-y^2})^2(d_{xy})^1$	8170	8550

Figure S9. Active orbitals of the ground state of **5** obtained by CASSCF(6,10) calculations. The five second d shell orbitals are omitted. The occupation number of each orbital e are listed near the label. The energies of $S = 2$ states predicted by CASSCF(6,10)/NEVPT2 calculations are listed in the table.

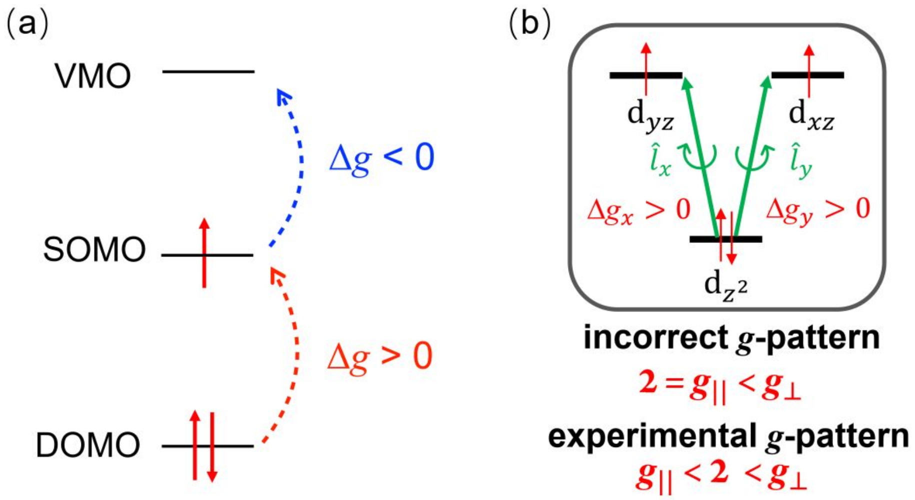

Figure S10. Sign of g-shifts for excitations from a doubly occupied molecular orbital (DOMO) to a singly occupied molecular orbital (SOMO) and from a SOMO to a virtual molecular orbital (VMO), respectively, derived by second-order perturbative treatment of SOC. (b) Sign of the g-shifts and (incorrect) g-pattern for complex **1** predicted by second-order perturbation theory. The transitions (green arrows) are used to indicate the orbital angular momentum operator ($\hat{l}_x$ and $\hat{l}_y$) responsible for their SOC with the ground state.

REFERENCES

1. Abo-Bakr, M.; Feikes, J.; Holldack, K.; Kuske, P.; Peatman, W. B.; Schade, U.; Wüstefeld, G.; Hübers, H. W., Brilliant, Coherent Far-Infrared (THz) Synchrotron Radiation. *Physical Review Letters* **2003,** *90*, 094801.
2. Nehrkorn, J.; Holldack, K.; Bittl, R.; Schnegg, A., Recent progress in synchrotron-based frequency-domain Fourier-transform THz-EPR. *J. Magn. Reson.* **2017,** *280*, 10-19.
3. Stoll, S.; Schweiger, A., EasySpin, a comprehensive software package for spectral simulation and analysis in EPR. *J. Magn. Reson.* **2006,** *178*, 42-55.
4. Chibotaru, L. F.; Ungur, L., Ab initio calculation of anisotropic magnetic properties of complexes. I. Unique definition of pseudospin Hamiltonians and their derivation. *J. Chem. Phys.* **2012,** *137*, 064112.
5. Tarrago, M.; Römelt, C.; Nehrkorn, J.; Schnegg, A.; Neese, F.; Bill, E.; Ye, S., Experimental and Theoretical Evidence for an Unusual Almost Triply Degenerate Electronic Ground State of Ferrous Tetraphenylporphyrin. *Inorg. Chem.* **2021,** *60*, 4966-4985.
